# #chatsafe 2.0. updated guidelines to support young people to communicate safely online about self-harm and suicide: A Delphi expert consensus study

**DOI:** 10.1371/journal.pone.0289494

**Published:** 2023-08-02

**Authors:** Jo Robinson, Pinar Thorn, Samuel McKay, Laura Hemming, Rikki Battersby-Coulter, Charlie Cooper, Maria Veresova, Angela Li, Nicola Reavley, Simon Rice, Michelle Lamblin, Jane Pirkis, Dan Reidenberg, Vicki Harrison, Jaelea Skehan, Louise La Sala

**Affiliations:** 1 Orygen, Parkville, Victoria, Australia; 2 Centre for Youth Mental Health, The University of Melbourne, Parkville, Victoria, Australia; 3 National Centre for Epidemiology and Population Health, Australian National University, Acton, Australian Capital Territory, Australia; 4 Centre for Mental Health, School of Population and Global Health, The University of Melbourne, Parkville, Victoria, Australia; 5 Suicide Awareness Voices of Education, Bloomington, Minnesota, United States of America; 6 Department of Psychiatry and Behavioral Sciences, Stanford University, Stanford, California, United States of America; 7 College of Health, Medicine and Wellbeing, University of Newcastle, Callaghan, New South Wales, Australia; The University of Adelaide, AUSTRALIA

## Abstract

**Introduction:**

Young people use social media to communicate about self-harm and suicide and this is associated with both potential risks and protective effects. The #chatsafe guidelines were originally developed in 2018 to equip young people to communicate safely online about suicide. They were shown to be safe, acceptable, and beneficial; however, they do not provide guidance on self-harm, and social media is constantly evolving. This study aimed to update the #chatsafe guidelines to reflect new evidence and current social media affordances, and to include guidance on self-harm.

**Methods:**

A Delphi expert consensus study was conducted, comprising six stages: 1) A systematic search of peer-reviewed and grey literature; 2) A series of roundtables with key stakeholders including social media companies, policymakers, and young people; 3) Questionnaire development; 4) Expert panel formation; 5) Data collection and analysis; and 6) Guideline development.

**Results:**

A total of 191 items were included in the new #chatsafe guidelines. These were organised into eight themes, which became the overarching sections of the guidelines: 1) General tips; 2) Creating self-harm and suicide content; 3) Consuming self-harm and suicide content; 4) Livestreams of self-harm and suicide acts; 4) Self-harm and suicide games, pacts, and hoaxes; 6) Self-harm and suicide communities; 7) Bereavement and communicating about someone who has died by suicide; and 8) Guidance for influencers.

**Discussion:**

The new guidelines include updated and new information on online communication about self-harm, livestreams, games, pacts, and hoaxes, as well as guidance for influencers. They will be disseminated via a national social media campaign and supported by a series of adult-facing resources. Given the acceptability of the original guidelines and the ubiquitous use of social media by young people, it is hoped that the new guidelines will be a useful resource for young people and adults alike, both in Australia and worldwide.

## Introduction

Youth suicide presents a significant public health problem [1]; it is the second-leading cause of death among young people worldwide, and in many countries rates of suicide appear to be increasing [2]. Rates of hospital presentations for self-harm are also increasing in young people [3], and, in addition to being problematic in its own right, self-harm is frequently associated with risk of future suicide [4]. Self-harm can be defined as a deliberate act of self-injury or self-poisoning, irrespective of motive or suicidal intent [5]. Indeed, self-harm is a complex behaviour and motives for engaging in it are varied [6–8]. Suicide refers to an action that a person takes to deliberately end their own life, which results in death.

Although the reasons for both youth suicide and self-harm are complex, much attention has been given to the potentially harmful role of social media [9]. Specifically, concerns exist regarding the potential for certain types of content to cause emotional distress and lead to ‘copycat’ acts by others [10–12]. However, social media is popular with young people. Young Australians spend over three hours per day on social media. This sometimes includes communicating with others about their own experiences of suicide and being exposed to suicide-related content created by others [13–15]. Our work, as well as the work of others, has identified many potential benefits of online communication about suicide. For example, it allows young people to create a sense of community, to seek help and provide help to others, and to grieve for people who have died by suicide. Additional benefits include its accessibility, non-stigmatizing nature, and capacity to deliver highly personalized content directly to an individual’s feed, which makes it an acceptable and attractive medium for communicating about sensitive topics and for seeking and providing help [14, 16]. Therefore, interventions that minimize the risks associated with social media while capitalizing on the benefits are needed.

Guidelines to improve communication by mainstream media are a widely accepted and cost-effective suicide prevention strategy [17–19], but these do not directly address the impacts of social media and are not designed with young people in mind. To address this, in 2018 we conducted a Delphi expert consensus study to inform the development of the #chatsafe guidelines, the world’s first guidelines designed to facilitate safe online communication about suicide among young people [20]. The guidelines contain five sections: 1) Things to consider before you post about suicide; 2) How to share your own story safely; 3) Communicating about someone who is affected by suicide; 4) Responding to someone who is suicidal; and 5) Communicating about someone who has died by suicide. They have been adapted for 12 countries [21], downloaded approximately 120,000 times, and cited in national and international policy documents and guidelines [22–24]. They were disseminated via a national co-designed suicide prevention campaign, which was co-designed with young people [25]. Social media content based on the guidelines were shown to be safe, acceptable, and beneficial in a pilot study [26]. The safety and effectiveness of social media content based on the original guidelines is now being tested in a randomised controlled trial with young people from across Australia (Database: ANZCTR, Trial registration number: ACTRN12622001397707) [27].

However, social media has rapidly evolved since the original guidelines were first published in 2018. For example, new platforms have emerged or become more popular (e.g., TikTok and BeReal), as have features such as livestreaming. Further, new research has been conducted which provides additional insights into both the harms and benefits of online communication about both self-harm and suicide [28, 29], meaning that the original guidelines were becoming outdated. Finally, the guidelines were exclusively focused on suicide and did not include any guidance on self-harm.

The aim of this study was to update and expand the #chatsafe guidelines, so that they better reflect both the evidence and the ways in which young people currently use social media to communicate about suicide and to include guidance on self-harm.

## Method

As with the original guidelines [20], this study employed the Delphi expert consensus method [30]. There were six stages: 1) A systematic search of peer-reviewed and grey literature; 2) A series of roundtables with key stakeholders including social media companies, policymakers, and young people; 3) Questionnaire development based on the included literature, findings from the roundtables, and team discussions; 4) Expert panel formation; 5) Data collection and analysis; and 6) Guideline development.

This study received approval from The University of Melbourne Human Research Ethics Committee (ID: 22728). All participants provided written informed consent.

### Systematic search of the literature

#### Inclusion criteria

Studies published in the peer reviewed or grey literature of any design and for any population were eligible for inclusion if they: 1) Focused on self-harm or suicide; 2) Focused on social media or other online environments; and 3) Focused on the nature of online communication about self-harm or suicide. Peer-reviewed articles had to be written in English, French, Spanish, or Russian (i.e., the languages spoken by the research team) to be included. Grey literature had to be written in English to be included.

#### Exclusion criteria

Studies and sources were excluded if they: 1) Focused on the relationship between social media, cyberbullying, or mental health broadly (as opposed to self-harm or suicide specifically); 2) Focused on machine learning or search engine data; 3) Focused on the development, or evaluation, of online interventions for self-harm or suicide (as opposed to online communication); 4) Did not contain statements relating to online communication about self-harm or suicide; 5) Involved actions that were mandated by law; or 6) Were conference abstracts, book chapters, editorials, or corrections.

#### Search strategy

The peer-reviewed literature search was conducted on 4 November 2021. We searched CINAHL, EMBASE, ERIC, Medline, PsycINFO, and Scopus for studies published from 1 January 2000. This start date was chosen to reflect the emergence and popularisation of social media [31].

Key search terms were developed by JR, LH, and PT in consultation with a university librarian. The following search terms were entered into each database title, key words, and abstracts: (social media or social network* or Instagram or YouTube or Myspace or Tumblr or Snapchat or Twitter or Facebook or Pinterest or TikTok or Flickr or LinkedIn or Skype or WhatsApp or Facetime or iMessage or Weibo or Reddit or Google+ or deviantart or livejournal or tagged or Orkut or blog* or chat or chatroom or online or forum or internet or web* or cyber* or electronic) AND ((deliberat* or self* or auto*) adj3 (destruct* or harm* or injur* or mutilat* or poison* or hurt* or cut* or inflict* or immolat*) or (SH or DSH or NSSI or parasuicid* or para suicid* or non-suicidal self injur* or overdos* or SIB or suicid*). RIS files were downloaded and imported into Covidence where all duplicates were automatically removed. Study titles and abstracts were single screened independently by five authors (SM, LH, RBC, MV, and AL). Due to the volume of studies, 90.0% of the total number of records were single screened, and 10.0% of records were independently double screened at both the title and abstract level by five of the authors (PT, SM, LH, MV and AL). Full texts were screened independently by three authors (LH, RBC, and MV). At each stage, discrepancies were resolved by discussion.

The grey literature search involved three components. First, the following databases were searched: APAIS-Health, Australian Policy Online, and ProQuest Dissertations and Theses Global (PQDT). As above, the search sought articles published between January 2000 and November 2021 and the same search terms were used; however, “adj3” was replaced with “AND” when searching APAIS-Health and “anywhere expect full next NOFT” was selected while searching PQDT. Study titles and abstracts and full texts were screened by one author (PT). Second, the first ten pages (i.e., up to the first 100 results) of google.com, google.com.au, google.ca, google.co.nz, and google.co.uk were searched. Two separate searches were conducted on the google search engines. Searches were limited to PDF documents. For the first search, we used the phrase: Suicide social media guideline. For the second search, we used the phrase: Self-harm social media guideline. Title links, URLs, and snippets, and PDF documents were screened by one author (PT). Finally, the ‘help centers’ or equivalent of Ask FM, Clubhouse, Deviant Art, Discord, Facebook, Instagram, Pinterest, Quora, Snapchat, TikTok, Tumblr, Twitch, Twitter, WhatsApp, and YouTube were searched and screened by one author (PT).

#### Data extraction and synthesis

Literature was searched for statements that included ideas about online communication about suicide or self-harm (“These recommendations emphasize that a focus on the celebrity’s life, i.e. what he or she contributed to the arts and to the society, is important and should be given priority instead on focusing on the suicidal act”), information that a person should know when communicating online about suicide or self-harm (“If someone threatens to take their own life, you should always take it seriously”), or actions a person should or should not take when communicating online about self-harm or suicide (“Do not post, upload, stream, or share: Suicide or self-harm games, dares, challenges, pacts, or hoaxes").

Relevant statements were extracted by PT and LH. Action items were generated from these statements (see Questionnaire Development).

### Round table consultations

Six roundtable consultations were conducted: three with social media companies (n = 7), two with Australian policy makers from the Department of Health (n = 14), and one with young people (n = 7; five identified as female, mean age = 20.7). Policy makers and representatives from the social media companies were known to the research team and were recruited via email by JR. Young people were recruited via a social media advertisement posted on the #chatsafe social media accounts. Young people who had previously participated in #chatsafe activities were notified of the participation opportunity via email. All young people were paid for their time. The roundtables were conducted between June and August 2022 by JR with assistance from LH, RBC, and LLS. The discussions were structured around the following questions: 1) From your perspective what are the challenges associated with online communication about self-harm and suicide?; 2) What more (if anything) could social media platforms and policy makers be doing to keep young people safe online; 3) To what extent do you think online safety is the responsibility of government, platforms and/or individuals; and 4) What else would you like to see in the new #chatsafe guidelines or associated resources?

Each session was audio recorded and transcribed verbatim. Although only the fourth question directly asked about the guidelines, all questions prompted spontaneous responses related to online communication about self-harm and suicide, therefore, transcripts in their entirety were searched for relevant statements and were extracted by PT, LH, and RBC. A qualitative analysis of this component of the project is underway and will be reported elsewhere.

### Questionnaire development

Statements were extracted from 149 peer-reviewed articles, 52 grey literature sources (including the original #chatsafe guidelines), and the six roundtables. They were then categorised thematically in a spreadsheet. A working group of three researchers (JR, PT, and LH), all of whom were experienced researchers in suicide prevention and the Delphi expert consensus method, omitted statements that contained repetitive information, and, where required, reworded statements for consistency and to contain one clear behavioural recommendation while preserving the original meaning (‘action item’). The statements from the original #chatsafe guidelines were all included in the questionnaire. The working group also created ‘action items’ based on their experience and feedback from young people on the original #chatsafe guidelines (e.g., items related to influencers). Assistance was provided, as required, by a fourth researcher (NR) who had expertise in the Delphi expert consensus method.

The Round 1 questionnaire contained 427 items and was organised into 10 sections (see Table 1). At the end of each section, panellists (see panel formation section below) were able to submit comments or suggestions to be included and rated in the Round 2 questionnaire. Two paid youth advisors (AD and EU) provided feedback on the survey to ensure that it was youth friendly.

**Table 1:** Sections included in the Delphi questionnaire and example items.

Section	Topic	Examples
**1**	Before posting online about self-harm or suicide	If posting or sharing about suicide / self-harm, young people should turn off the comment function Young people should create a plan for if they become upset or troubled by posts that they have shared or seen
**2**	Sharing your own thoughts, feelings, or experiences	Young people should not post, share, or respond to content that provides links to pro-suicide sites or forums Young people should provide an accompanying trigger or content warning to graphic or descriptive content
**3**	Communicating about someone else	If writing or sharing a post about someone who is suspected to have died by suicide, young people should not tag the individual If writing or sharing a post about someone who has died by suicide, young people should post only what they know to be true.
**4**	Responding to someone who may be at risk of suicide or self-harm	If a young person comes across content that suggests a person may be thinking about self-harm or suicide, they should inform a trusted adult If a young person decides to respond to a post that concerns them, they should respond without judgement, assumptions or interruptions
**5**	Groups	If establishing a closed group or forum, young people should moderate all comments for harmful or unsafe content If establishing a closed group or forum, young people should create a ‘Terms of Use’ that outlines the rules for participating in the page or group
**6**	Suicide games, hoaxes, and pacts	Young people should not post, upload, stream or share self-harm or suicide games Young people should report suicide pacts to the police
**7**	Self-harm	Young people should not post or share graphic photos relating to self-harm Young people should post information about the possible reasons for self-harm
**8**	Humour	Young people should use humour to discuss suicide / self-harm Young people should not use humour to belittle others
**9**	Livestreams	Young people should not post a live stream of a self-harm or suicide act Young people should not take screenshots of content from a livestream of suicide / self-harm
**10**	Influencers	Young people considered ‘influencers’ should provide information about sources of support / resources Young people considered ‘influencers’ should not portray themselves as an expert in suicide prevention

To develop the Round 2 questionnaire, the working group reviewed the panellists’ comments, and, if they were original ideas that had not been included in the Round 1 questionnaire, they were added to the Round 2 questionnaire. The Round 2 questionnaire also included items that did not reach consensus to be included or excluded from the guidelines in the Round 1 questionnaire and required re-rating. Overall panel ratings obtained in Round 1 were reported alongside each of these items. The Round 2 questionnaire comprised 118 items to be rated for the guidelines. Again, there was an opportunity for panellists to provide final comments.

This questionnaire also included a series of complementary questions to be rated by the panels on what they thought social media companies and policymakers could be doing to improve online safety. Examples of these items included: “Social media companies should provide support to all employees and volunteers working with self-harm or suicide content”. The findings from this additional component of the survey will be analysed and reported separately.

### Panel formation and recruitment

Two expert panels were recruited. One comprised suicide prevention professionals and one comprised young people.

Panels of around 20 members produce stable findings in Delphi studies, with one study reporting that 23 experts per panel produced stability in response characteristics [30, 32]. In total, our panels comprised 103 members. Of these, 29 were professionals and 74 were young people.

#### Professional panel

The professional panellists were identified via the studies included in the peer-reviewed literature and sources included in the grey literature. Potential panellists were invited to participate via email. They were eligible for inclusion if they: 1) Were aged at least 18 years; 2) Were an expert on self-harm or suicide (e.g., research, teach, or treat self-harm or suicide; conducted and published research on self-harm or suicide and social media; or contributed to guidelines on communication about self-harm or suicide); and 3) Were proficient in English.

The professional panel included PhD students (13.8%), postdoctoral researchers (10.3%), a senior research fellow (3.5%), senior lecturers/assistant professors/readers (13.8%), associate professors (24.1%), professors (24.1%), and those in other roles such as advisors and funders (6.9%). One panellist did not report their role. One fifth of professional panellists also worked as clinicians (20.7%). Most held a doctoral qualification (86.2%), with the remaining holding a masters (10.3%) or honours (3.5%) degree. They resided in a variety of countries including Australia (20.7%), UK (17.24%), USA (17.4%), Austria (6.9%), New Zealand (6.9%), and 3.5% each from Canada, China, Estonia, France, Germany, Ghana, Hong Kong, South Africa, and Spain.

Professional panellists were not asked to report their age; therefore, it is unknown if the professional panel included young people (i.e., those up to 25 years old inclusive. For example, the PhD students or early career researchers).

#### Youth panel

Youth panel members were recruited from Instagram via organic and paid advertisements. Young people were eligible to be included if they: 1) Were aged between 15 and 25 years inclusive; 2) Lived in Australia; 3) Were proficient in English; and 4) Had seen, communicated about, or wanted to communicate online about self-harm or suicide.

The mean age of youth panellists was 21.30 years (*SD* = 2.54, range = 17–25). Over one half of youth panellists were female (59.5%), one quarter were trans or gender diverse (25.7%), and 14.7% were male. Over one half identified as LGBTIQA+ (54.1%), and almost one quarter (23.0%) came from a culturally and/or linguistically diverse background [33]. Most had living or lived experience of self-harm or suicide (the type of experience was not specified; 82.4%) and/or had supported someone who was self-harming or suicidal (63.5%); a smaller proportion had been bereaved by suicide (18.9%). Most youth panellists were employed (casual employment 40.5%; part time employment 13.5%; full time employment 16.2%; unemployed 29.7%). The education level was diverse: 13.2% had not completed Year 12; 40.5% had completed Year 12; 21.6% had completed a certificate or TAFE; 12.2% had completed and obtained a bachelor’s degree; 9.5% had completed and obtained an honours degree, graduate certificate, or graduate diploma; and 2.7% had completed and obtained a master’s degree.

### Delphi questionnaires

Data were collected in two questionnaire rounds via the web-based survey platform, Qualtrics. Panellists were asked to indicate if each item should be included in the new guidelines for young people on safe online communication about self-harm and suicide. For pragmatic reasons, three response options were used: “Yes”, “Unsure”, and “No”. These were defined for participants as ’Yes’ = agree, include this item in the guidelines; ’Unsure’ = unsure if this item should be in the guidelines; and ’No’ = disagree, exclude this item from the guidelines. The middle point was included because of the sensitive subject matter and because neutrality or uncertainty was considered a valid option. Different rating scales including the yes/no scale are used for Delphi consensus studies, but ultimately the process ends with a dichotomous result and consensus is generally not reached for all items due to pragmatic reasons [34–37]. The questionnaires were divided into nine sections representing common topic themes. Panellists were able to provide free text comments and suggestions at the end of each section.

#### Delphi questionnaire data analysis

The survey data were analysed in Microsoft Excel. Pearson’s *r* was calculated to assess agreement for the proportion items endorsed for inclusion in the guidelines within and between the youth and professional panels.

#### Round 1 questionnaire

The Round 1 questionnaire comprised 427 items. Like the previous #chatsafe study, items that received a “yes” response from 80.0% or more of both the panels were eligible to be included in the guidelines. Items were re-rated as part of Round 2 if 80.0% or more of only one of the panels provided a “yes” response to the item, or if 70–79.9% of both panels provided a “yes” response for the item. Items that did not meet the above criteria were excluded from the Round 2 questionnaire and guidelines. The working group reviewed all panellists feedback provided in Round 1, and new ideas not already captured in Round 1 were included as a new item in the second questionnaire.

#### Round 2 questionnaire

The Round 2 questionnaire included 90 items to be re-rated as well as 28 new items that were generated based on the panel feedback provided in Round 1. All panellists were provided with the Round 1 results for all re-rate items and were asked to consider this information when re-rating items during the Round 2 questionnaire. Items that received a “yes” response from 80.0% or more of both panels were eligible to be included in the guidelines. All other items were excluded.

### Guideline development

The research team combined all included items that contained similar content and PT wrote them into prose for the final guidelines with assistance from JR and the wider research team. Given the audience, the authors used lay language to ensure the guidelines were accessible and acceptable to young people. The final draft was reviewed by three paid youth advisors (MG, AD, EU) who provided feedback on language and style. Careful consideration was given to ensuring that the final guidelines were true to the original meaning of the questionnaire items whilst still being coherent and easy to read. The draft guidelines were then provided to panellists for final feedback and endorsement. Three professional panellists requested minor changes, which were implemented (e.g., adding a few additional lines of psychoeducation, linking back to language tips, and clarifying and simplifying terms).

## Results

### Systematic search results

#### Peer-reviewed literature

**Fig 1:**
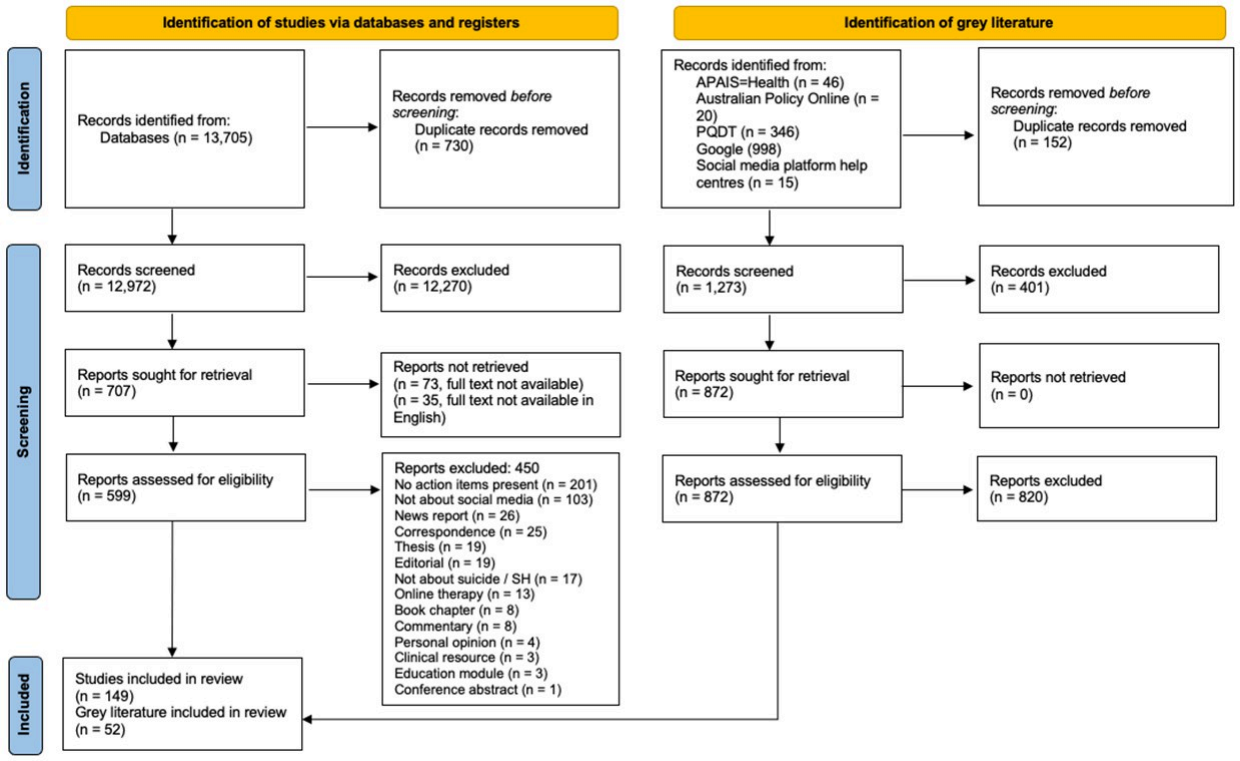
Prisma flow diagram.

In total, 13,705 articles were retrieved via database searching. Following initial screening, 599 full-text articles were retrieved, of which, 149 met our inclusion criteria (see Fig 1).

#### Grey literature

In total, 46 sources were retrieved from APAIS-Health, and, following initial screening none of which were eligible for inclusion. In total, 20 sources were retrieved from Australian Policy Online, of these, three were duplicates, and, following initial screening none were eligible for inclusion. In total, 346 sources were retrieved from PQDT, of these, three were duplicates. Following initial screening, five full-text theses were retrieved; all five met inclusion criteria and were included. In total, 998 sources were retrieved from the Google searches, of these, 146 were duplicates. Following full-text screening, 32 PDF documents met inclusion criteria, and all were included. In total, 15 sources were retrieved from the social media platform’s help centres or equivalent webpages; all of which were included.

#### Questionnaire results

**Fig 2:**
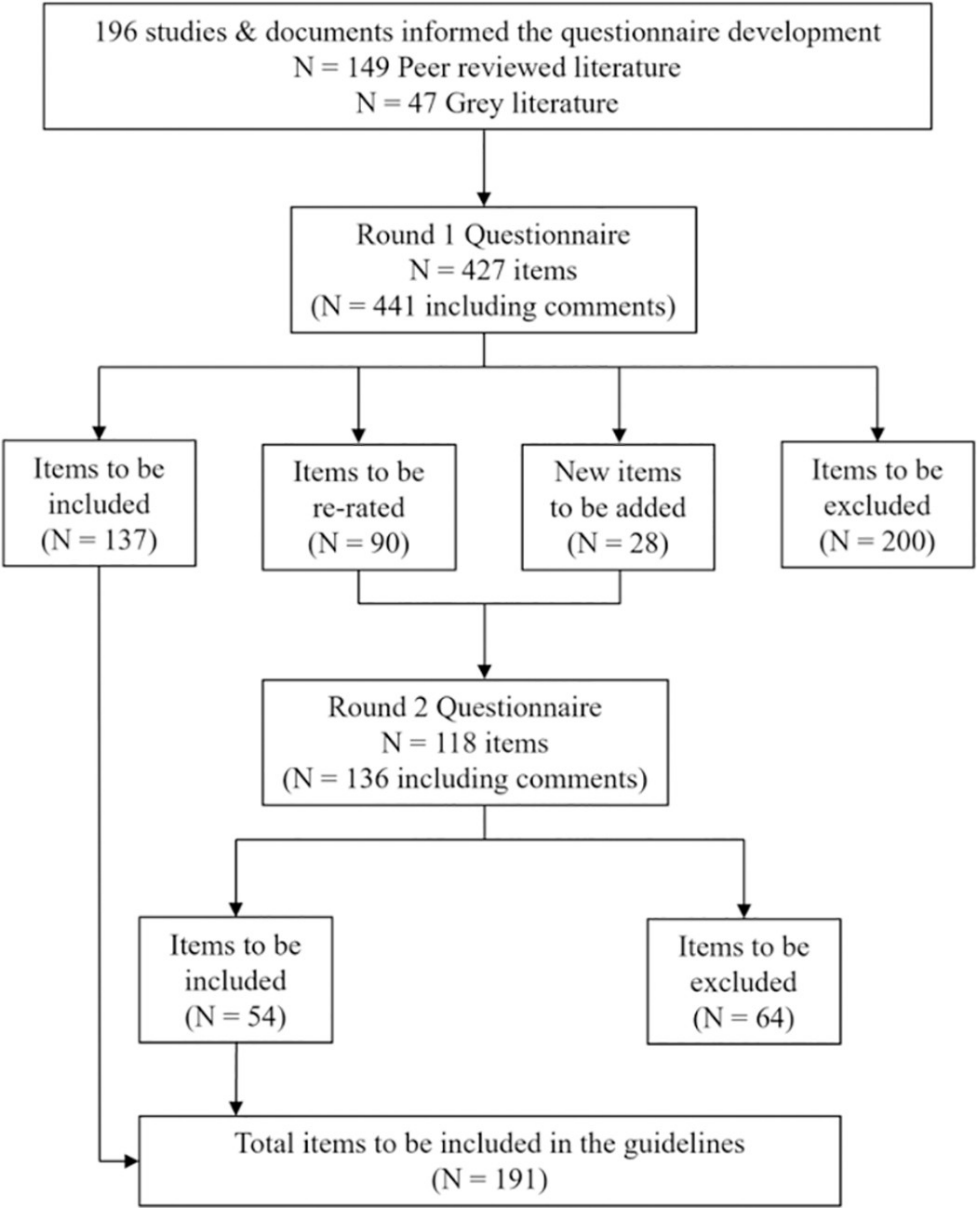
Flow chart of included studies and questionnaire items by Delphi round.

The participation rate of panellists completing the two rounds of questionnaires was 65.1% (58.1% for youth and 82.8% for professionals; See Table 2). Fig 2 shows the number of items included, excluded, and rerated during the two questionnaire rounds. The panels both rated 454 items in total (427 items from Round 1 and 28 new items in Round 2 based on participant feedback in Round 1). Overall, 191 items of the 454 items (186 from the original questionnaire and five feedback items provided by panellists) were rated as “yes” for inclusion in the guidelines by at least 80.0% of both panels (S1 File shows the individual item results for the two questionnaire rounds). The correlation between the two panels was strong in both Round 1 (*r* = 0.90, *p* < .001) and Round 2 (*r* = 0.81, *p* < .001).

**Table 2:** Round 1 and Round 2 questionnaire participation rates by panel.

	Round 1 (n)	Round 2 (n)	Completion (%)
**Youth**	74	43	58.1%
**Professional**	29	24	82.8%
**Total**	103	67	65.1%

Despite the general agreement, there were some categories and individual questionnaire items where the youth and professional panels had a higher proportion of disagreement. These items did not meet consensus, and so were not included in the final guidelines. For example, items that received notably higher scores (>20.0%) by the professional panel focused on not posting content related to self-harm and scars at all. In contrast, the youth panel were more likely to endorse items that placed an increased level of responsibility on the person posting or interacting with the content: By monitoring or limiting post responses; posting trigger warnings; gaining permission from relevant parties such as family or the affected person before posting about suicide and suicide related behaviours; undertaking risk assessments or providing support to others; or actively seeking to discredit or reduce the risk of suicide hoaxes or self-harm behaviour. It is important to note that most discrepant items did not meet the threshold for inclusion in either panel, limiting their impact on the results.

The final guidelines are organised into the following eight sections: 1) General tips; 2) Creating self-harm and suicide content; 3) Consuming self-harm and suicide content; 4) Livestreams of self-harm and suicide acts; 4) Self-harm and suicide games, pacts, and hoaxes; 6) Self-harm and suicide communities; 7) Bereavement and communicating about someone who has died by suicide; and 8) Guidance for influencers. Where appropriate, self-harm and suicide guidance was combined; however, 18 items were specifically related to interactions about self-harm regardless of motive (e.g., “avoid comparing self-harm scars or injuries” and “if appropriate, urge the person to seek professional help for any inquiries”). The guidelines are publicly available via the following website: https://www.orygen.org.au/chatsafe.

## Discussion

This study used the Delphi expert consensus method to update and expand the original #chatsafe guidelines, so that they better reflect extant evidence and the current ways in which young people use social media to communicate about suicide and to include guidance on self-harm. Whilst the guidelines have been written with young people in mind, they may be equally beneficial to adults.

### How the new guidelines compare to the original ones

The most salient difference between the original and new guidelines is the inclusion of guidance on how to safely communicate online about self-harm. Self-harm is prevalent among young people and highly stigmatised (even in health services), which can deter formal help-seeking [38–41], meaning that young people may be more likely to experience and be exposed to self-harm compared to suicide and to turn to social media platforms for help and support. Several reviews have shown that online communication about self-harm is nuanced and associated with potential benefits and potential harms [29, 42–47]. Consequently, young people need guidance on how to safely create and consume online content related to self-harm, and thus #chatsafe was expanded to include self-harm. In Round 1, some items that were excluded were highly endorsed by the youth panel, but not the professional panel. Unsurprisingly, professionals did not endorse young people taking a very active role when providing support (e.g., “Post first-aid advice on how to care for [self-harm] injuries”). This reluctance to endorse intervention was also seen in items related to fear appeals such as posting about the risks of self-harm (e.g., scars and permanent injuries). Indeed, in Round 1, most discrepancies between the panels related to what young people should not do when responding to self-harm content. Six out of the 10 items had to be rerated in Round 2. Eventually, only two self-harm specific items did not reach consensus: Posting advice on how people can self-harm without injuring themselves too badly and not using humour when someone has engaged in self-harm behaviour.

Another change between the original and new guidelines is the inclusion of a specific section for influencers (also known as social media content creators). To the best of our knowledge, although some resources do exist for people posting about their lived experience, no specific and publicly available evidence-informed guidance on how to communicate safely about self-harm or suicide exists for this group. This is despite their considerable followings, and, in some cases, frequent posts about mental health including their own self-harm and suicide experiences. This can be problematic in multiple ways. For example, they may share their own self-harm or suicide experiences in unsafe ways, they may find themselves in a dialogue with vulnerable young people and not feel equipped to respond safely, or they may provide advice that is not evidence-based or even potentially harmful. Further complicating matters, often influencer content includes paid ads in disguise. This is something that social media companies are aware is a problem, and some have started to deliver training to influencers to help them talk about health related topics more responsibly [48]. Congruently, the Australian Therapeutic Goods Administration (as well as other international policymakers) have implemented strict provisions relating to influencer marketing such as rules on ad disclosures [49]. Although codes of practice currently relate to goods and services, their existence speaks to the broader issue of consumers making choices based on influencer testimonials that are not always factual or evidence based, indicating that guidance is also needed for areas such as mental health. Indeed, traditional media and journalists have codes and considerations for reporting on suicide-related content [50]. However, social media has made it possible for anyone to create, post, and share content, which is not regulated in the same manner.

The new guidelines also include sections on suicide games, pacts, hoaxes, and livestreams, all of which have caused significant concern in recent times. For example, a recent and well-known challenge that allegedly encouraged people to engage in increasingly serious acts of self-harm that ultimately led to suicide, reportedly went viral on social media, and led to significant concerns about the safety of younger users. Although some have concluded that the game itself was likely a hoax, and much of the communication about it involved users warning others about the potential harms, it received significant attention from the mainstream media, and the potential for distress and potentially harmful impacts remained [51–53]. In the current study, both panels agreed that content relating to a suicide game should not be posted or shared, and if users encounter this type of content, they should report it to the relevant platform.

Livestreams operate differently to other types of content including pre-recorded videos. These are real-time and unedited videos of people engaging in acts of self-harm, some of which result in suicide, delivered to consumers without any significant delays or time gaps. By that fact, unlike pre-recorded videos, the content creator may be more impulsive, and the consumers can engage with the content creator in real time and may not have any warning of what is about to be streamed. Again, these have received significant attention in the mainstream media [54–56]. They also present challenges for the social media platforms themselves, as on the one hand they have the potential to spread quickly and expose large numbers of people to a real-time suicide, but on the other hand if the post remains live there is the potential for the platforms (or viewers) to intervene [57]). In the current study, however, both panels agreed that, despite the potential for intervention in real time as opposed to retrospectively, acts of self-harm or suicide should not be livestreamed. This is reflected in the new guidelines, which specifically recommend that people do not engage with livestreams of self-harm or suicide acts and if people encounter them, they should avoid interacting with them, rather they should report them directly to the platform and emergency services (if appropriate).

There were also some items that were in the original guidelines that did not reach consensus for inclusion in the new guidelines. For example, directly asking someone if they are suicidal was excluded by both panels. Including trigger or content warnings ahead of an online post about suicide or self-harm was also excluded. A recent meta-analysis concluded that content or trigger warnings (albeit not related specifically to self-harm or suicide-related content) neither reduce engagement with, nor negative reactions to, sensitive content [58]. However, trigger warnings are still commonly used and as such guidance may still be useful for young people. For this reason, we decided to include some information on this, as well as explaining why it is not unsafe to ask someone if they are suicidal in the guidelines, but we presented in such a way that it was clear that they were not endorsed by the panels.

### Implementing the guidelines

As before, the new guidelines will be housed on the #chatsafe website and will be disseminated via a national social media campaign that will be co-designed with young people across the country. They will also be supported by a series of adult-facing resources including for bereaved communities, family members, and educators. Funding permitting, they will be translated for an international audience as was the case with the original guidelines [21].

Until recently, there have been few (if any) studies that have actively involved young people in the co-design of a universal suicide prevention intervention [59, 60] and limited data exists regarding the effectiveness of suicide prevention public health campaigns. However, social media metrics (e.g., engagement, impressions, and reach) from the original campaign demonstrates that this approach can reach large numbers of young people quickly. Further, data from the evaluation of the co-design process showed that it was not just safe and acceptable to involve young people in developing a suicide prevention intervention, but it also had inherent benefits. For example, young people reported feeling better able to communicate safely online about suicide after participating in the co-design workshops, as well as better able to identify and respond to someone at risk of suicide [25]. Similarly, in a pilot study, social media content based on the original guidelines showed that participants’ willingness to intervene online against suicide as well as perceived self-efficacy, confidence, and safety when communicating on social media about suicide increased [26].

### Strengths and limitations

This study has several strengths and limitations. A key strength is the innovative and highly translational nature of the guidelines. A further strength has been the involvement of young people, both with and without lived experience of self-harm and suicide. As noted above, despite growing evidence demonstrating the benefits of involving people with lived experience in research [61], this is still rarely done in youth suicide research [59, 60]. However, our previous studies have shown that involving young people can be both safe and beneficial for young people, [25] and, as such it was considered essential to have young people involved in this study from start to finish.

In terms of limitations, the nature of the Delphi methodology means that some items that may be important did not reach consensus, possibly because they may not be appropriate in most situations, and, as a result, are not included in the final guidelines. This includes items that were endorsed when developing the original guidelines. This means that there are areas for which guidance is lacking (e.g., the use of content warnings and how to ask someone online if they are feeling suicidal). There are some groups who were under-represented in the youth panel, such as adolescents, Aboriginal and Torres Strait Islander peoples and people from diverse cultural backgrounds. This means that the guidelines may be less applicable to these populations. Additionally, some groups were overrepresented, for example, many of our participants identified as female or gender and sexually diverse; however, LGBTIQA+ young people are overrepresented in suicide statistics. Moreover, as noted above, most youth panellists had living or lived experience of self-harm or suicide; therefore, parts of the guidelines may not resonate with the general youth population. However, again, these young people are most likely to engage in communication about self-harm and suicide and are at risk of repeat self-harm and future suicide and need assistance. A final limitation applies to Delphi studies more broadly and relates to the fact that studies such as this, that generate evidence via a process of consensus are useful when it is hard to generate the evidence using more robust study designs [30]. However, that said it remains important to then evaluate the guidelines produced to ensure that they are effective in achieving their aims. The original #chatsafe guidelines have been evaluated in a pre-test post-test study with promising results and are now being tested in a randomised controlled trial [27].

## Conclusions

This study updated the original #chatsafe guidelines, the world’s first evidence-informed guidelines developed to support young people to communicate safely online about suicide. The new guidelines include key updates including guidance on communicating about self-harm, games, pacts, livestreams, and guidance for influencers. As previously, they will be disseminated via a national social media campaign co-designed with young people and supported by a suite of adult-facing resources, which will be housed on the #chatsafe website as well as the safety centres of our industry partners. Given the popularity of the original guidelines and the increasing use of social media by young people, it is hoped that the new guidelines will also prove to be a useful resource for young people and adults alike, not just in Australia but worldwide.

## Supporting information

S1 File Questionnaire data. (DOCX)
