# Supplementary material for: #chatsafe 2.0. updated guidelines to support young people to communicate safely online about self-harm and suicide: A Delphi expert consensus study
Source: PLoS One. 2023 Aug 2;18(8):e0289494. doi: 10.1371/journal.pone.0289494 (PMC10395901; doi:10.1371/journal.pone.0289494)
Supplement: S1 File — (DOCX) [file pone.0289494.s001.docx]

**S1 File. Questionnaire data.**

**Delphi questionnaire Round 1 results.**

| **Q #** | **Question** | **Professionals** | **Young People** | **Item result** |
| --- | --- | --- | --- | --- |
|  | **Young people should post or share images, photos, video content or animations:** |  |  |  |
| 1 | monitor the post regularly for unsafe or potentially harmful comments | 48.28% | 85.14% | Rerate |
| 2 | include phone numbers or links to appropriate help services in the post (e.g. helplines, local suicide prevention services, local emergency services if appropriate) when monitoring is not an option | 79.31% | 95.95% | Rerate |
| 3 | turn off the comment function | 13.79% | 22.97% | Exclude |
| 4 | edit content before posting | 65.52% | 58.11% | Exclude |
| 5 | create a specific anonymous account specifically for this purpose | 3.45% | 13.51% | Exclude |
| 6 | restrict access to their post (e.g. not make it available to those under 18 years of age) if appropriate to that platform | 37.93% | 43.24% | Exclude |
| 7 | young people should not create a specific anonymous account to do so | 37.93% | 32.43% | Exclude |
| 8 | Young people should create a plan for what to do if they become upset or troubled by posts that they have either shared or seen | 86.21% | 94.59% | Include |
|  | **If young people are feeling upset or overwhelmed by suicide / self-harm content, they should:** |  |  |  |
| 9 | talk to someone about how they are feeling | 93.10% | 100.00% | Include |
| 10 | take a break (e.g. physically step away, log out of social media) | 96.55% | 95.95% | Include |
| 11 | take control of the content they see (e.g. hide posts, unfollow content) | 86.21% | 97.30% | Include |
| 12 | delete social media friends whose posts they find upsetting | 37.93% | 59.46% | Exclude |
|  | **If a young person is having thoughts of suicide, they should** |  |  |  |
| 13 | post only about their thoughts or feelings, but not actions or behaviours | 34.48% | 52.70% | Exclude |
| 14 | post openly about their thoughts, feelings and/or behaviours | 13.79% | 20.27% | Exclude |
| 15 | post only about their thoughts or feelings, but not actions or behaviours | 37.93% | 52.70% | Exclude |
| 16 | post openly about their thoughts, feelings and/or behaviours | 10.34% | 21.62% | Exclude |
| 17 | post about this on social media | 10.34% | 16.22% | Exclude |
| 18 | post about this on social media | 13.79% | 18.92% | Exclude |
|  | **If a young person is sharing their own thoughts or experiences of suicide, they should** |  |  |  |
| 19 | include information on support services (e.g. links to suicide prevention or counselling helpline) | 75.86% | 85.14% | Rerate |
| 20 | include information on support services in a prominent position (e.g. at the beginning of the post) | 62.07% | 74.32% | Exclude |
| 21 | encourage help-seeking | 82.76% | 93.24% | Include |
| 22 | only include messages of hope and recovery | 17.24% | 18.92% | Exclude |
| 23 | include messages that convey hope and recovery as well as hopeless messages (e.g. things will never get better) | 37.93% | 35.14% | Exclude |
| 24 | highlight the people, places or things that supported their recovery (e.g. things that have helped them cope) | 89.66% | 93.24% | Include |
| 25 | include experiences of positive help-seeking | 89.66% | 93.24% | Include |
| 26 | focus on the context that has led them to feel suicidal or like self-harming rather than how they would suicide or self-harm | 68.97% | 68.92% | Exclude |
|  | **If a young person is sharing their own thoughts or experiences of self-harm, they should:** |  |  |  |
| 27 | include information on support services (e.g. links to suicide prevention or counselling helpline) | 72.41% | 85.14% | Rerate |
| 28 | include information on support services in a prominent position (e.g. at the beginning of the post) | 58.62% | 77.03% | Exclude |
| 29 | encourage help-seeking | 82.76% | 94.59% | Include |
| 30 | only include messages of hope and recovery | 17.24% | 21.62% | Exclude |
| 31 | include messages that convey hope and recovery as well as hopeless messages (e.g. things will never get better) | 37.93% | 37.84% | Exclude |
| 32 | highlight the people, places or things that supported their recovery (e.g. things that have helped them cope) | 89.66% | 93.24% | Include |
| 33 | include experiences of positive help-seeking | 89.66% | 93.24% | Include |
| 34 | focus on the context that has led them to feel suicidal or like self-harming rather than how they would suicide or self-harm | 68.97% | 72.97% | Exclude |
|  | **If a young person is sharing their own thoughts or experiences of suicide, they should not:** |  |  |  |
| 35 | include content that blames other people | 62.07% | 67.57% | Exclude |
| 36 | include content that makes others feel responsible for their safety | 58.62% | 82.43% | Rerate |
| 37 | ask others to keep it secret | 62.07% | 68.92% | Exclude |
| 38 | plan their own suicide or self-harm on social media | 72.41% | 83.78% | Rerate |
| 39 | use emojis to indicate means or methods of suicide / self-harm | 68.97% | 72.97% | Exclude |
|  | **If a young person is sharing their own thoughts or experiences of self-harm, they should not:** |  |  |  |
| 40 | include content that blames other people | 62.07% | 71.62% | Exclude |
| 41 | include content that makes others feel responsible for their safety | 58.62% | 85.14% | Rerate |
| 42 | ask others to keep it secret | 62.07% | 68.92% | Exclude |
| 43 | plan their own suicide or self-harm on social media | 72.41% | 85.14% | Rerate |
| 44 | use emojis to indicate means or methods of suicide / self-harm | 68.97% | 71.62% | Exclude |
|  | **If a young person is sharing their suicide, recovery story they should:** |  |  |  |
| 45 | share recovery process in steps | 62.07% | 68.92% | Exclude |
| 46 | report progress | 65.52% | 70.27% | Exclude |
| 47 | report lapses / relapses | 55.17% | 51.35% | Exclude |
| 48 | share accomplishments | 93.10% | 95.95% | Include |
| 49 | report how they have resisted urges of suicide / self-harm | 89.66% | 82.43% | Include |
| 50 | post about positive help-seeking experiences (e.g. treatment experiences) | 100.00% | 94.59% | Include |
| 51 | emphasise that ‘recovery’ can be different for everyone and is a process with ups and downs | 93.10% | 97.30% | Include |
|  | **If a young person is sharing their self-harm, recovery story they should:** |  |  |  |
| 52 | share recovery process in steps | 65.52% | 68.92% | Exclude |
| 53 | report progress | 65.52% | 70.27% | Exclude |
| 54 | report lapses / relapses | 55.17% | 50.00% | Exclude |
| 55 | share accomplishments | 93.10% | 95.95% | Include |
| 56 | report how they have resisted urges of suicide / self-harm | 89.66% | 82.43% | Include |
| 57 | post about positive help-seeking experiences (e.g. treatment experiences) | 100.00% | 94.59% | Include |
| 58 | emphasise that ‘recovery’ can be different for everyone and is a process with ups and downs | 93.10% | 97.30% | Include |
|  | **If a young person feels that another person has helped them in terms of their suicide / self-harm, they should thank them:** |  |  |  |
| 59 | publicly | 10.34% | 10.81% | Exclude |
| 60 | privately | 48.28% | 82.43% | Rerate |
|  | **Young people should not post content that:** |  |  |  |
| 61 | provides links to pro-suicide sites or forums | 89.66% | 86.49% | Include |
| 62 | promotes or encourages suicide or self-harm | 93.10% | 94.59% | Include |
| 63 | is intended to shock or disgust others | 75.86% | 77.03% | Rerate |
| 64 | provides information or instructions for engaging in suicide / self-harm | 93.10% | 94.59% | Include |
| 65 | co-ordinates suicide pacts | 89.66% | 94.59% | Include |
| 66 | provides information about suicide pacts | 82.76% | 78.38% | Rerate |
| 67 | includes suggestive signs or emojis (e.g. things that can be used to harm yourself or take your life) | 79.31% | 77.03% | Rerate |
| 68 | include concerning hashtags (e.g. popular hashtags that may be trending that encourage or promote suicide / self-harm) | 86.21% | 81.08% | Include |
| 69 | mocks or makes fun of victims or survivors of suicide / self-harm | 89.66% | 93.24% | Include |
| 70 | contains a suicide note or message | 72.41% | 77.03% | Rerate |
|  | **Young people should not share content that:** |  |  |  |
| 71 | provides links to pro-suicide sites or forums | 89.66% | 87.84% | Include |
| 72 | promotes or encourages suicide or self-harm | 93.10% | 95.95% | Include |
| 73 | is intended to shock or disgust others | 79.31% | 77.03% | Rerate |
| 74 | provides information or instructions for engaging in suicide / self-harm | 93.10% | 93.24% | Include |
| 75 | co-ordinates suicide pacts | 89.66% | 94.59% | Include |
| 76 | provides information about suicide pacts | 82.76% | 78.38% | Rerate |
| 77 | includes suggestive signs or emojis (e.g. things that can be used to harm yourself or take your life) | 79.31% | 79.73% | Rerate |
| 78 | include concerning hashtags (e.g. popular hashtags that may be trending that encourage or promote suicide / self-harm) | 82.76% | 83.78% | Include |
| 79 | mocks or makes fun of victims or survivors of suicide / self-harm | 89.66% | 93.24% | Include |
| 80 | contains a suicide note or message | 72.41% | 74.32% | Rerate |
|  | **Young people should not respond to content that:** |  |  |  |
| 81 | provides links to pro-suicide sites or forums | 51.72% | 60.81% | Exclude |
| 82 | promotes or encourages suicide or self-harm | 55.17% | 72.97% | Exclude |
| 83 | is intended to shock or disgust others | 51.72% | 60.81% | Exclude |
| 84 | provides information or instructions for engaging in suicide / self-harm | 58.62% | 71.62% | Exclude |
| 85 | co-ordinates suicide pacts | 62.07% | 79.73% | Exclude |
| 86 | provides information about suicide pacts | 62.07% | 66.22% | Exclude |
| 87 | includes suggestive signs or emojis (e.g. things that can be used to harm yourself or take your life) | 58.62% | 63.51% | Exclude |
| 88 | include concerning hashtags (e.g. popular hashtags that may be trending that encourage or promote suicide / self-harm) | 58.62% | 68.92% | Exclude |
| 89 | mocks or makes fun of victims or survivors of suicide / self-harm | 55.17% | 71.62% | Exclude |
| 90 | contains a suicide note or message | 48.28% | 50.00% | Exclude |
|  | **If a young person has previously had thoughts of suicide / self-harm but not acted on them, they should:** |  |  |  |
| 91 | post about the things that stopped them from attempting suicide (e.g. loved ones, consequences of previous attempts). | 68.97% | 66.22% | Exclude |
|  | **If a young person is sharing their thoughts or experiences of suicide / self-harm because they want help, they should:** |  |  |  |
| 92 | explicitly state that they need support | 79.31% | 82.43% | Rerate |
| 93 | explicitly state that they want an urgent response | 55.17% | 62.16% | Exclude |
| 94 | use the word ‘help’ in their post | 62.07% | 64.86% | Exclude |
|  | **If a young person is sharing their thoughts or experiences of suicide / self-harm but they do not want help, they should:** |  |  |  |
| 95 | explicitly state that they are expressing their thoughts, feelings and experiences simply to make them feel better (i.e. venting) | 65.52% | 81.08% | Rerate |
| 96 | explicitly state that they want their thoughts and feelings to be validated (e.g. by other people who have experienced similar thoughts or feelings) | 48.28% | 68.92% | Exclude |
|  | **If a young person is sharing their thoughts or experiences of suicide / self-harm for their own benefit (e.g. not to interact with others), they should:** |  |  |  |
| 97 | keep that account private | 44.83% | 64.86% | Exclude |
| 98 | keep that content private (e.g. change audience settings so nobody can view it) | 65.52% | 71.62% | Exclude |
|  | **Trigger / content warnings: When posting a warning, young people should use the following language:** |  |  |  |
| 99 | 'trigger warning' | 75.86% | 85.14% | Rerate |
| 100 | 'TW' | 27.59% | 68.92% | Exclude |
| 101 | 'content warning' | 75.86% | 87.84% | Rerate |
| 102 | 'CW' | 27.59% | 59.46% | Exclude |
| 103 | 'not safe for work' | 20.69% | 36.49% | Exclude |
| 104 | 'NSFW' | 6.90% | 32.43% | Exclude |
| 105 | 'disclaimer' | 24.14% | 54.05% | Exclude |
|  | **Young people should provide an accompanying trigger or content warning to:** |  |  |  |
| 106 | graphic or descriptive content | 89.66% | 97.30% | Include |
| 107 | only content that contains images, photos or videos | 34.48% | 35.14% | Exclude |
| 108 | any post relating to suicide / self-harm (e.g. even if there are no images) | 58.62% | 90.54% | Rerate |
| 109 | images of people who have died by suicide | 72.41% | 67.57% | Exclude |
|  | **When posting a warning, young people should include the warning:** |  |  |  |
| 110 | at the beginning of a text post | 86.21% | 97.30% | Include |
| 111 | in a post before posting the content (e.g. warning, and then post the content in a comment below) | 55.17% | 72.97% | Exclude |
| 112 | in the content caption / description box (e.g. Instagram captions / YouTube description box) | 68.97% | 78.38% | Exclude |
| 113 | in the first image of the content (e.g. in the case of a series of images being posted) | 65.52% | 94.59% | Rerate |
|  | **When posting a warning, young people should include:** |  |  |  |
| 114 | just the words ‘content / trigger warning’ | 13.79% | 27.03% | Exclude |
| 115 | information on what is contained in the post (e.g. warning - this post contains information on suicide / self-harm / images of somebody that has died by suicide) | 79.31% | 93.24% | Rerate |
| 116 | the option for users to view the content anyway | 51.72% | 85.14% | Rerate |
| 117 | a link to, or information about, available support services | 93.10% | 95.95% | Include |
|  | **Posting visual content: When posting about suicide / self-harm, young people should post:** |  |  |  |
| 118 | photos / images alone | 0.00% | 6.76% | Exclude |
| 119 | videos alone | 0.00% | 8.11% | Exclude |
| 120 | text alone | 44.83% | 45.95% | Exclude |
| 121 | photos / images / videos and text | 20.69% | 44.59% | Exclude |
|  | **Young people should not post or share images, photos, video content or animations:** |  |  |  |
| 122 | that depict a method of suicide / self-harm | 89.66% | 87.84% | Include |
| 123 | that depict the location of a suicide / self-harm | 82.76% | 77.03% | Rerate |
| 124 | of wounds | 86.21% | 75.68% | Rerate |
| 125 | of injuries | 79.31% | 75.68% | Rerate |
| 126 | of self-harm equipment or accessories (clean or used / soiled) | 86.21% | 86.49% | Include |
| 127 | of active self-harming behaviour (e.g. self-harm in progress) | 93.10% | 93.24% | Include |
| 128 | of healing scars | 55.17% | 22.97% | Exclude |
| 129 | of healed scars | 44.83% | 10.81% | Exclude |
| 130 | of grieving friends or family members of those who have died by suicide without their permission | 79.31% | 79.73% | Rerate |
| 131 | of memorial services for those who have died by suicide | 41.38% | 27.03% | Exclude |
| 132 | of the person’s body (e.g. photographs from a funeral viewing) | 89.66% | 75.68% | Rerate |
| 133 | which blurs images of methods, but also includes details of methods in accompanying text | 79.31% | 72.97% | Rerate |
|  | **Young people should post or share images, photos, video content or animations:** |  |  |  |
| 134 | of healed scars | 10.34% | 64.86% | Exclude |
| 135 | of the person’s body (e.g. photographs from a funeral viewing) if the image is blurred or covered | 0.00% | 13.51% | Exclude |
| 136 | which focus on wounds but with a narrative of progress or hope in the accompanying text | 10.34% | 36.49% | Exclude |
| 137 | which focus on scars but with a narrative of hope in the accompanying text (e.g. tattoos of hope over scars) | 31.03% | 75.68% | Exclude |
| 138 | which focus on wounds but with a narrative of help-seeking in the accompanying text | 13.79% | 36.49% | Exclude |
| 139 | which focus on scars but with a narrative of help-seeking in the accompanying text | 24.14% | 64.86% | Exclude |
|  | **Young people should not post or share videos of:** |  |  |  |
| 140 | During a live attempt at suicide - the lead-up to a suicide | 72.41% | 85.14% | Rerate |
| 141 | During a live attempt at suicide - suicide attempts in progress | 72.41% | 89.19% | Rerate |
| 142 | During a live attempt at suicide - suicide rescue footage | 65.52% | 77.03% | Exclude |
| 143 | When someone has already died by suicide - the lead-up to a suicide | 75.86% | 77.03% | Rerate |
| 144 | When someone has already died by suicide - suicide attempts in progress | 75.86% | 87.84% | Rerate |
| 145 | When someone has already died by suicide - suicide rescue footage | 62.07% | 75.68% | Exclude |
| 146 | If posting images / photos / videos relating to suicide / self-harm, young people should use captions (e.g. text under the visual content) to provide context | 68.97% | 89.19% | Rerate |
|  | **Young people should not:** |  |  |  |
| 147 | post suicide goodbye images / photos / videos | 75.86% | 62.16% | Exclude |
| 148 | compliment images / photos /videos of wounds posted by others or suggest techniques | 89.66% | 91.89% | Include |
|  | **Language to use when posting about suicide / self-harm online: When posting about suicide, young people should not use language that:** |  |  |  |
| 149 | describes suicide as criminal or sinful (e.g. committed suicide) | 100.00% | 78.38% | Rerate |
| 150 | describes suicide as a ‘solution’ to problems, life stressors or mental health difficulties | 96.55% | 87.84% | Include |
| 151 | describes suicide as glamourous, romantic or appealing | 96.55% | 93.24% | Include |
| 152 | trivialises suicide or makes it seem less complex than it really is (e.g. don’t blame one event or imply suicide was the result of a single cause) | 96.55% | 87.84% | Include |
| 153 | sensationalises suicide (e.g. don’t provide links to sensational ‘clickbait’) | 96.55% | 91.89% | Include |
| 154 | is judgmental and reinforces myths, stigma and stereotypes (e.g. suicide is for cowards or a cry for help) | 96.55% | 90.54% | Include |
| 155 | provides detailed information about the suicide or suicide attempt (e.g. methods, location) | 96.55% | 86.49% | Include |
| 156 | describes suicide as a desirable outcome (e.g. successful, unsuccessful or failed attempts) | 96.55% | 85.14% | Include |
|  | **When posting about someone affected by thoughts of suicide or self-harm: If a young person is concerned about someone who has experienced suicidal or self-harm thoughts or feelings, they should not:** |  |  |  |
| 157 | post anything they would not say directly to them in-person | 72.41% | 83.78% | Rerate |
| 158 | post anything without their permission | 62.07% | 90.54% | Rerate |
|  | **If writing or sharing a post about someone who has died by suicide, young people should:** |  |  |  |
| 159 | - Death of someone the young person knows - post only what they know to be true | 82.76% | 91.89% | Include |
| 160 | - Death of someone the young person knows - encourage others to post only what they know to be true (e.g. avoid speculating about the death) | 89.66% | 85.14% | Include |
| 161 | - Death of someone the young person knows - ask online friends / followers to demonstrate respect and empathy when posting about someone who has died by suicide | 89.66% | 91.89% | Include |
| 162 | - Death of someone the young person knows - ask online friends / followers to consider how their comments might impact others before posting about someone who has died by suicide | 86.21% | 94.59% | Include |
| 163 | - Death of someone the young person knows - dispel myths or misinformation | 75.86% | 75.68% | Rerate |
| 164 | - Death of someone the young person knows - explicitly state that they have received permission from the family in their post | 58.62% | 82.43% | Rerate |
| 165 | - Death of someone the young person knows - focus on the life of the person rather than the death | 62.07% | 78.38% | Exclude |
| 166 | - Death of someone the young person knows - allow other people to add comments | 31.03% | 45.95% | Exclude |
| 167 | - Death of someone the young person knows - moderate comments | 58.62% | 93.24% | Rerate |
| 168 | - Death of someone the young person knows - be clear about whether people can contact them for support or not | 79.31% | 89.19% | Rerate |
| 169 | - Death of someone the young person knows - only post suicide notes or letters with sufficient context | 10.34% | 43.24% | Exclude |
| 170 | - Death of someone the young person knows - provide links to available support services | 86.21% | 94.59% | Include |
| 171 | - Death of someone the young person knows - share / post content which educates others (e.g. about suicide / self-harm prevention) | 82.76% | 91.89% | Include |
| 172 | - Death of someone the young person knows - tag the person who has died by suicide | 3.45% | 13.51% | Exclude |
| 173 | - Death of a celebrity or public figure - post only what they know to be true | 79.31% | 83.78% | Rerate |
| 174 | - Death of a celebrity or public figure - encourage others to post only what they know to be true (e.g. avoid speculating about the death) | 82.76% | 82.43% | Include |
| 175 | - Death of a celebrity or public figure - ask online friends / followers to demonstrate respect and empathy when posting about someone who has died by suicide | 82.76% | 90.54% | Include |
| 176 | - Death of a celebrity or public figure - ask online friends / followers to consider how their comments might impact others before posting about someone who has died by suicide | 82.76% | 91.89% | Include |
| 177 | - Death of a celebrity or public figure - dispel myths or misinformation | 75.86% | 68.92% | Exclude |
| 178 | - Death of a celebrity or public figure - explicitly state that they have received permission from the family in their post | 27.59% | 40.54% | Exclude |
| 179 | - Death of a celebrity or public figure - focus on the life of the person rather than the death | 62.07% | 77.03% | Exclude |
| 180 | - Death of a celebrity or public figure - allow other people to add comments | 24.14% | 47.30% | Exclude |
| 181 | - Death of a celebrity or public figure - moderate comments | 51.72% | 85.14% | Rerate |
| 182 | - Death of a celebrity or public figure - be clear about whether people can contact them for support or not | 72.41% | 81.08% | Rerate |
| 183 | - Death of a celebrity or public figure - only post suicide notes or letters with sufficient context | 10.34% | 41.89% | Exclude |
| 184 | - Death of a celebrity or public figure - provide links to available support services | 86.21% | 94.59% | Include |
| 185 | - Death of a celebrity or public figure - share / post content which educates others (e.g. about suicide / self-harm prevention) | 82.76% | 89.19% | Include |
| 186 | - Death of a celebrity or public figure - tag the person who has died by suicide | 3.45% | 13.51% | Exclude |
|  | **If writing or sharing a post about someone who has died by suicide, young people should not:** |  |  |  |
| 187 | - Death of someone the young person knows - tag the individual who has died by suicide | 58.62% | 52.70% | Exclude |
| 188 | - Death of someone the young person knows - speculate or form theories about a person’s thoughts or feelings leading up to the suicide | 75.86% | 86.49% | Rerate |
| 189 | - Death of someone the young person knows - speculate or form theories about why the person took their life | 72.41% | 83.78% | Rerate |
| 190 | - Death of someone the young person knows - post or share information that they are unsure of or that they know is inaccurate | 82.76% | 90.54% | Include |
| 191 | - Death of someone the young person knows - post without getting permission from the deceased's next of kin | 58.62% | 74.32% | Exclude |
| 192 | - Death of someone the young person knows - allow others to post comments | 17.24% | 31.08% | Exclude |
| 193 | - Death of someone the young person knows - moderate comments | 17.24% | 28.38% | Exclude |
| 194 | - Death of someone the young person knows - allow comments that minimise others feelings about the death | 65.52% | 77.03% | Exclude |
| 195 | - Death of someone the young person knows - allow comments that indicate the poster is going to attempt suicide (e.g. 'I will join you soon') | 65.52% | 82.43% | Rerate |
| 196 | - Death of someone the young person knows - post or share suicide notes or letters | 72.41% | 66.22% | Exclude |
| 197 | - Death of someone the young person knows - re-post stories or links about suicide attempts or death | 48.28% | 59.46% | Exclude |
| 198 | - Death of someone the young person knows - re-post stories or links about suicide attempts or death until the information is confirmed | 55.17% | 79.73% | Exclude |
| 199 | - Death of someone the young person knows - encourage others to imitate the suicide act | 82.76% | 87.84% | Include |
| 200 | - Death of someone the young person knows - share content that discusses the suicide in an unsafe way (e.g. describes methods, contains graphic images) | 79.31% | 83.78% | Rerate |
| 201 | - Death of someone the young person knows - post or share too many posts about the suicide back to back | 72.41% | 68.92% | Exclude |
| 202 | - Death of a celebrity or public figure - tag the individual who has died by suicide | 58.62% | 51.35% | Exclude |
| 203 | - Death of a celebrity or public figure - speculate or form theories about a person’s thoughts or feelings leading up to the suicide | 72.41% | 81.08% | Rerate |
| 204 | - Death of a celebrity or public figure - speculate or form theories about why the person took their life | 72.41% | 79.73% | Rerate |
| 205 | - Death of a celebrity or public figure - post or share information that they are unsure of or that they know is inaccurate | 79.31% | 86.49% | Rerate |
| 206 | - Death of a celebrity or public figure - post without getting permission from the deceased's next of kin | 37.93% | 40.54% | Exclude |
| 207 | - Death of a celebrity or public figure - allow others to post comments | 17.24% | 31.08% | Exclude |
| 208 | - Death of a celebrity or public figure - moderate comments | 13.79% | 25.68% | Exclude |
| 209 | - Death of a celebrity or public figure - allow comments that minimise others feelings about the death | 65.52% | 75.68% | Exclude |
| 210 | - Death of a celebrity or public figure - allow comments that indicate the poster is going to attempt suicide (e.g. 'I will join you soon') | 65.52% | 79.73% | Exclude |
| 211 | - Death of a celebrity or public figure - post or share suicide notes or letters | 68.97% | 63.51% | Exclude |
| 212 | - Death of a celebrity or public figure - re-post stories or links about suicide attempts or death | 48.28% | 54.05% | Exclude |
| 213 | - Death of a celebrity or public figure - re-post stories or links about suicide attempts or death until the information is confirmed | 55.17% | 74.32% | Exclude |
| 214 | - Death of a celebrity or public figure - encourage others to imitate the suicide act | 82.76% | 86.49% | Include |
| 215 | - Death of a celebrity or public figure - share content that discusses the suicide in an unsafe way (e.g. describes methods, contains graphic images) | 79.31% | 81.08% | Rerate |
| 216 | - Death of a celebrity or public figure - post or share too many posts about the suicide back to back | 72.41% | 71.62% | Rerate |
| 217 | **When posting about someone who is suspected to have died by suicide:** If writing or sharing a post about someone who is suspected to have died by suicide, young people should not tag the individual who may have died by suicide | 79.31% | 63.51% | Exclude |
|  | **If a young person comes across a post that shares a suicide story in a way they consider to be unsafe, they should:** |  |  |  |
| 218 | encourage the poster to reword their message so it is safer and repost | 68.97% | 83.78% | Rerate |
| 219 | ask the poster to remove content that they find upsetting | 41.38% | 63.51% | Exclude |
|  | **Coming across content that suggests a person may be thinking about suicide / self-harm: If a young person comes across content that suggests a person may be thinking about suicide or self-harm, they should:** |  |  |  |
| 220 | always take it seriously | 89.66% | 95.95% | Include |
| 221 | inform a trusted adult | 86.21% | 83.78% | Include |
| 222 | inform a friend | 27.59% | 52.70% | Exclude |
| 223 | seek professional advice (e.g. a phone or online support service, or health professional) | 68.97% | 89.19% | Rerate |
| 224 | report the content to the relevant platform | 75.86% | 68.92% | Exclude |
| 225 | read other comments to see if the person has already received help | 65.52% | 77.03% | Exclude |
| 226 | remove or hide comments where possible (e.g. where the comments are in response to their own post) | 20.69% | 50.00% | Exclude |
| 227 | ask them directly if they are at risk of suicide / self-harm | 55.17% | 67.57% | Exclude |
|  | **If a young person comes across content that suggests a person may be thinking about suicide or self-harm, they should not:** |  |  |  |
| 228 | ignore the post | 68.97% | 71.62% | Exclude |
| 229 | share the post | 75.86% | 81.08% | Rerate |
| 230 | respond if they feel overwhelmed | 72.41% | 68.92% | Exclude |
|  | **If the person is not at immediate risk of suicide, young people should:** |  |  |  |
| 231 | encourage the person to seek professional help | 86.21% | 94.59% | Include |
| 232 | ask the person ‘what can I do to help?’ | 65.52% | 86.49% | Rerate |
| 233 | ask the person if they would like them to provide contact details for local support services | 96.55% | 91.89% | Include |
| 234 | respect the person’s wishes if they tell them they do not want help | 68.97% | 62.16% | Exclude |
| 235 | check in with the person later, if they feel comfortable | 89.66% | 97.30% | Include |
|  | **If the person is at immediate risk of suicide, young people should:** |  |  |  |
| 236 | encourage the person to call emergency services (if appropriate) | 100.00% | 97.30% | Include |
| 237 | contact the person’s family or someone in their social network who can check on them | 89.66% | 94.59% | Include |
| 238 | call emergency services immediately (and if appropriate tell the person they’re doing this) | 75.86% | 79.73% | Rerate |
| 239 | contact a trusted adult and let them know what is happening | 96.55% | 87.84% | Include |
| 240 | contact a friend and let them know what is happening | 27.59% | 52.70% | Exclude |
| 241 | report the post to the platform | 75.86% | 63.51% | Exclude |
| 242 | take screenshots of content so they can share this with emergency services e.g. if police (if appropriate) | 82.76% | 85.14% | Include |
|  | **The following items relate to additional things that young people could do if they want to interact with the person posting. If a young person decides to respond, they should:** |  |  |  |
| 243 | use emojis or reactions that show compassion (e.g. hugs, care) | 51.72% | 67.57% | Exclude |
| 244 | provide details for support (e.g. phone lines, online support services) | 89.66% | 93.24% | Include |
| 245 | tag others who they think may be able to help | 20.69% | 16.22% | Exclude |
| 246 | use a standard template to respond (e.g. that they have created themself or that they have found elsewhere) | 10.34% | 27.03% | Exclude |
| 247 | use memes as a way to cheer the person up | 10.34% | 20.27% | Exclude |
| 248 | redirect the person to inspirational videos or quotes | 3.45% | 13.51% | Exclude |
| 249 | tell the person that they care | 75.86% | 87.84% | Rerate |
| 250 | tell the person that they will be there for them no matter what | 20.69% | 60.81% | Exclude |
| 251 | respond without judgement, assumptions or interruptions | 89.66% | 97.30% | Include |
| 252 | let the person explain their thoughts, feelings and experiences in their own words | 89.66% | 98.65% | Include |
| 253 | ask the person how they would prefer them to respond (e.g. in private, in public, online, offline) | 89.66% | 94.59% | Include |
| 254 | respond using the medium that the responder feels most comfortable with (e.g. messages, comment, phone call) | 75.86% | 91.89% | Rerate |
| 255 | look at the person’s posts to acknowledge their feelings and specify exactly why they are worried about them | 68.97% | 90.54% | Rerate |
| 256 | give the person their full attention | 65.52% | 90.54% | Rerate |
| 257 | try and assess the level of risk (e.g. ask if they have a suicide / self-harm plan and/or means to enact that) | 41.38% | 87.84% | Rerate |
| 258 | set boundaries regarding the type of support they are able to offer and when they are able to offer it | 89.66% | 95.95% | Include |
| 259 | cautiously check whether the person has tried to harm themselves previously (e.g. sensitively ask the person if they have ever hurt themselves on purpose) | 48.28% | 78.38% | Exclude |
| 260 | cautiously check whether the person has further suicide plans | 58.62% | 83.78% | Rerate |
| 261 | ask other friends (on or off social media) to build a team of support | 31.03% | 51.35% | Exclude |
| 262 | if appropriate / possible, visit the individual in person | 41.38% | 79.73% | Exclude |
| 263 | if not possible to visit the person, consider reaching out to people who live locally | 44.83% | 67.57% | Exclude |
| 264 | offer to ‘sit’ with the person virtually in real time | 65.52% | 90.54% | Rerate |
|  | **If a young person decides to respond to a post that concerns them, they should not:** |  |  |  |
| 265 | respond in such a way that encourages or endorses suicide / self-harm behaviour (e.g. asking for a joint suicide, encouraging somebody to take their life, giving advice on how to suicide / self-harm, complimenting / admiring techniques) | 100.00% | 91.89% | Include |
| 266 | use emojis or reactions that positively reinforce or ridicule (e.g. laughing, or ‘likes’) | 86.21% | 87.84% | Include |
| 267 | compare the person’s distress to something bigger (e.g. say that there are worse things happening in the world) | 96.55% | 89.19% | Include |
| 268 | tag others in their reply | 79.31% | 74.32% | Rerate |
| 269 | use a standard template to respond (e.g. that they have created themself or that they have found elsewhere) | 55.17% | 43.24% | Exclude |
| 270 | accuse the person of lying | 100.00% | 87.84% | Include |
| 271 | offer support if they do not feel able or comfortable to do so | 93.10% | 74.32% | Rerate |
| 272 | try and assess the level of risk (e.g. ask if the person has a plan, means to enact the plan) | 27.59% | 25.68% | Exclude |
|  | **Moderating: If running a closed group or forum, young people should** |  |  |  |
| 273 | moderate comments for potentially harmful or unsafe content | 93.10% | 98.65% | Include |
| 274 | approve all posts before they can appear publicly in the group | 68.97% | 78.38% | Exclude |
| 275 | allow people to post freely | 3.45% | 18.92% | Exclude |
| 276 | turn comment functions off | 6.90% | 9.46% | Exclude |
| 277 | report content to platform that is beyond the abilities of those moderating | 82.76% | 89.19% | Include |
| 278 | not moderate comments | 0.00% | 5.41% | Exclude |
|  | **If running a closed group or forum, moderators should:** |  |  |  |
| 279 | check in with members who have not been active in the group for a while | 44.83% | 47.30% | Exclude |
| 280 | announce publicly if a group member dies by suicide | 10.34% | 17.57% | Exclude |
| 281 | if announcing a suicide death, identify the person properly (e.g. not just their screen name details | 20.69% | 22.97% | Exclude |
| 282 | delete comments that violate terms of use (see Q103) | 86.21% | 95.95% | Include |
| 283 | remove or suspend members that violate terms of use (see Q103) | 75.86% | 86.49% | Rerate |
|  | **If running a closed group or forum, moderators should not:** |  |  |  |
| 284 | announce if a group member dies by suicide | 34.48% | 44.59% | Exclude |
| 285 | if announcing a suicide death, identify the person | 51.72% | 41.89% | Exclude |
|  | **Support: If setting up a closed group or forum, young people should:** |  |  |  |
| 286 | advise group members that this is not a replacement for professional support | 100.00% | 100.00% | Include |
| 287 | provide support contact details visibly on the page at all times | 96.55% | 97.30% | Include |
| 288 | only provide support contact details at times when the group is not monitored | 17.24% | 17.57% | Exclude |
| 289 | ask potential group members to complete an application before joining the group | 37.93% | 70.27% | Exclude |
|  | **If setting up a closed group or forum, young people should not:** | 20.69% | 12.16% | Exclude |
| 290 | ask potential group members to complete an application before joining the group |  |  |  |
| 291 | require group members to share evidence of suicide / self-harm as part of their application to join the group | 82.76% | 75.68% | Rerate |
| 292 | **Terms of use: If setting up a closed group or forum, young people should** create a ‘terms of use’ that outlines the rules for participating in the page or group | 96.55% | 100.00% | Include |
|  | **Terms of use should:** |  |  |  |
| 293 | encourage group members to look after their own wellbeing | 96.55% | 95.95% | Include |
| 294 | make it clear that graphic details about suicide are not allowed | 93.10% | 95.95% | Include |
| 295 | outline the approval process for comments and feedback from other group members | 96.55% | 97.30% | Include |
| 296 | outline the process for responding to or addressing comments that suggest a group member is at risk of suicide / self-harm | 96.55% | 97.30% | Include |
| 297 | explain when and how group members can report potentially harmful content to the platform | 96.55% | 98.65% | Include |
| 298 | explain when and how group members can report potentially harmful content to group moderators | 96.55% | 100.00% | Include |
| 299 | provide a contact form or other contact details for group members to report potentially unsafe or concerning content | 82.76% | 98.65% | Include |
| 300 | explain that violation of the terms of use will result in comments being deleted | 82.76% | 98.65% | Include |
| 301 | outline the steps that will be taken to remove comments or group members who violate the terms of use | 89.66% | 97.30% | Include |
| 302 | if suspending, deleting or blocking a group member, let the person know why their behaviour is concerning | 93.10% | 100.00% | Include |
| 303 | explain that group members cannot copy or forward content without permission from the original poster | 82.76% | 95.95% | Include |
|  | **Groups or pages to memorialise those who have died by suicide:** |  |  |  |
| 304 | If somebody has died by suicide, young people should create a memorial page or group | 6.90% | 9.46% | Exclude |
| 305 | If somebody has died by suicide, young people should not create a memorial page or group | 24.14% | 17.57% | Exclude |
|  | **If creating a group to memorialise somebody who has died by suicide, young people should:** |  |  |  |
| 306 | create a distinct memorial thread or chat to bring the group together to mourn | 44.83% | 48.65% | Exclude |
| 307 | create a temporary memorial page | 37.93% | 24.32% | Exclude |
| 308 | create a permanent memorial page | 10.34% | 18.92% | Exclude |
| 309 | create a memorial video to be shared on social media | 10.34% | 16.22% | Exclude |
| 310 | explain that group members must include details of the person who died by suicide in their posts | 3.45% | 6.76% | Exclude |
| 311 | make clear to users that there is likely to be detailed information and images of people who have died | 51.72% | 82.43% | Rerate |
|  | **Groups or pages for self-harm: If a young person comes across a group or page for self-harm, they should:** |  |  |  |
| 312 | report self-harm groups to the platform | 68.97% | 77.03% | Exclude |
| 313 | report self-harm groups to the e-Safety Commission or similar body | 58.62% | 72.97% | Exclude |
| 314 | report self-harm groups to the police | 24.14% | 35.14% | Exclude |
|  | **Young people should not:** |  |  |  |
| 315 | start a self-harm group | 68.97% | 77.03% | Exclude |
| 316 | join a self-harm group | 62.07% | 71.62% | Exclude |
| 317 | assign roles to people to co-ordinate a self-harm group (e.g. to bring tools for self-harm) | 89.66% | 85.14% | Include |
| 318 | host or engage in self-harm online parties | 100.00% | 87.84% | Include |
| 319 | self-harm to remain part of an online group | 96.55% | 89.19% | Include |
|  | **Suicide / self-harm games: Young people should:** |  |  |  |
| 320 | create or share content that raises awareness of the game | 10.34% | 27.03% | Exclude |
| 321 | create or share content that criticises the game | 20.69% | 48.65% | Exclude |
| 322 | report the game to the platform | 93.10% | 98.65% | Include |
| 323 | report the game to e-Safety Commission or similar body | 86.21% | 95.95% | Include |
| 324 | report the game to the police | 41.38% | 68.92% | Exclude |
|  | **Young people should not:** |  |  |  |
| 325 | post, upload, stream or share suicide or self-harm games / dares / trends | 96.55% | 93.24% | Include |
| 326 | share content showing participation in or instructions for suicide / self-harm challenges | 96.55% | 93.24% | Include |
| 327 | interact with posts advertising suicide / self-harm games (e.g. comment, like or react) | 82.76% | 85.14% | Include |
| 328 | ask others, or seek partners, to engage in suicide / self-harm games | 96.55% | 94.59% | Include |
|  | **Suicide hoaxes: Young people should:** |  |  |  |
| 329 | create or share content that raises awareness of the hoax | 24.14% | 50.00% | Exclude |
| 330 | create or share content that criticises the hoax | 24.14% | 50.00% | Exclude |
| 331 | report the hoax to the platform | 89.66% | 98.65% | Include |
| 332 | report the hoax to e-Safety Commission or similar body | 79.31% | 90.54% | Rerate |
| 333 | report the hoax to the police | 31.03% | 56.76% | Exclude |
|  | **Young people should not:** |  |  |  |
| 334 | share or re-post content about suicide hoaxes | 86.21% | 72.97% | Rerate |
| 335 | interact with posts about suicide hoaxes (e.g. comment, like or react) | 86.21% | 72.97% | Rerate |
|  | **Suicide pacts: Young people should:** |  |  |  |
| 336 | report suicide pacts to the platform | 93.10% | 93.24% | Include |
| 337 | report suicide pacts to e-Safety Commission or similar body | 86.21% | 86.49% | Include |
| 338 | report suicide pacts to the police | 65.52% | 79.73% | Exclude |
|  | **Young people should not:** |  |  |  |
| 339 | start a suicide pact | 96.55% | 91.89% | Include |
| 340 | join a suicide pact | 96.55% | 89.19% | Include |
| 341 | assign roles to people to co-ordinate a suicide pact (e.g. to bring tools for suicide) | 96.55% | 91.89% | Include |
|  | **Posting about self-harm** |  |  |  |
| 342 | When posting about their own experiences of self-harm, young people should request suggestions for alternative coping strategies | 58.62% | 72.97% | Exclude |
|  | **When posting about their own experiences of self-harm, young people should not post or share:** |  |  |  |
| 343 | before and after pictures of self-harm | 93.10% | 83.78% | Include |
| 344 | graphic photos relating to self-harm (e.g. methods of self-harm) | 96.55% | 93.24% | Include |
| 345 | pictures of “scar tattoos” or “self-harm stickers” (e.g. in the shape of scars) | 75.86% | 54.05% | Exclude |
| 346 | content that includes negative self-talk about self-harm (e.g. I’m a failure for relapsing) | 65.52% | 59.46% | Exclude |
| 347 | content that talks about feelings associated with self-harm that may be construed as positive (e.g. relief, release of pressure, etc.) | 68.97% | 45.95% | Exclude |
| 348 | step by step self-harm guides | 96.55% | 91.89% | Include |
| 349 | content that encourages others to imitate self-harm acts | 100.00% | 91.89% | Include |
| 350 | content about another person’s self-harm without their permission | 93.10% | 89.19% | Include |
|  | **Responding to posts about self-harm: When responding to a post about self-harm, young people should:** |  |  |  |
| 351 | suggest alternatives to self-harm (e.g. self-soothing strategies, a virtual hope box) | 72.41% | 90.54% | Rerate |
| 352 | suggest distractions from self-harm (e.g. exercise, taking a hot bath, listening to soothing music) | 72.41% | 93.24% | Rerate |
| 353 | suggest wider coping strategies to deal with the underlying problem(s) (e.g. speak to friends) | 75.86% | 95.95% | Rerate |
| 354 | if appropriate, urge the poster to seek professional help for physical wellbeing (e.g. wounds not healing properly) | 93.10% | 95.95% | Include |
| 355 | positively reinforce and praise milestones and progress of others | 79.31% | 93.24% | Rerate |
| 356 | remind people that ‘lapses’ or ‘relapses’ can be part of the process | 79.31% | 89.19% | Rerate |
| 357 | encourage the person to keep trying to refrain from self-harm if they have lapsed or relapsed | 75.86% | 90.54% | Rerate |
| 358 | challenge the person to see how much time they can refrain from self-harm if they have lapsed or relapsed | 24.14% | 50.00% | Exclude |
| 359 | recommend online self-harm forums and groups | 34.48% | 50.00% | Exclude |
| 360 | ask the person if they’re suicidal | 37.93% | 40.54% | Exclude |
|  | **When either posting about, or responding to posts about, self-harm, young people should:** |  |  |  |
| 361 | provide inspirational messages | 34.48% | 43.24% | Exclude |
| 362 | post first-aid advice on how to care for injuries | 41.38% | 67.57% | Exclude |
| 363 | post advice on how people can reduce risks while self-harming (i.e., harm minimisation) | 34.48% | 70.27% | Exclude |
| 364 | post about the risks associated with self-harm (e.g. accidental death / infection) | 41.38% | 64.86% | Exclude |
| 365 | post about the potential permanency of self-harm (e.g. scars, enduring harm, disabilities) | 41.38% | 60.81% | Exclude |
| 366 | post information about the possible reasons for self-harm | 31.03% | 45.95% | Exclude |
| 367 | post educational information to help people who are self-harming (e.g. how they can stop) | 79.31% | 90.54% | Rerate |
|  | **When either posting about, or responding to posts about, self-harm, young people should not:** |  |  |  |
| 368 | normalise self-harm behaviour (e.g. post that self-harm is an acceptable coping behaviour, fixes things or makes you feel better) | 93.10% | 79.73% | Rerate |
| 369 | celebrate self-harm | 96.55% | 87.84% | Include |
| 370 | encourage others to share photos of their self-harm wounds | 96.55% | 83.78% | Include |
| 371 | compare self-harm scars or injuries | 93.10% | 87.84% | Include |
| 372 | compete with other people online who self-harm | 93.10% | 91.89% | Include |
| 373 | self-harm to show admiration or support for someone online | 93.10% | 91.89% | Include |
| 374 | post advice on how people can self-harm without injuring themselves too badly | 86.21% | 72.97% | Rerate |
| 375 | post advice on more severe methods of self-harm | 100.00% | 91.89% | Include |
|  | **Young people should use humour in these circumstances:** |  |  |  |
| 376 | to discuss suicide | 10.34% | 13.51% | Exclude |
| 377 | to discuss self-harm | 10.34% | 13.51% | Exclude |
| 378 | when sharing their own story | 17.24% | 36.49% | Exclude |
| 379 | when responding to others stories | 0.00% | 1.35% | Exclude |
| 380 | when someone has died by suicide | 3.45% | 1.35% | Exclude |
| 381 | when someone has self-harmed | 3.45% | 0.00% | Exclude |
| 382 | in text posts | 6.90% | 10.81% | Exclude |
| 383 | in video / image posts | 6.90% | 8.11% | Exclude |
| 384 | only if they have lived experience | 20.69% | 43.24% | Exclude |
|  | **Young people should not use humour in these circumstances:** |  |  |  |
| 385 | to discuss suicide | 58.62% | 47.30% | Exclude |
| 386 | to discuss self-harm | 58.62% | 48.65% | Exclude |
| 387 | to belittle others | 82.76% | 86.49% | Include |
| 388 | when sharing their own story | 20.69% | 28.38% | Exclude |
| 389 | when responding to others stories | 65.52% | 64.86% | Exclude |
| 390 | when someone has died by suicide | 75.86% | 82.43% | Rerate |
| 391 | when someone has self-harmed | 75.86% | 78.38% | Rerate |
| 392 | in text posts | 34.48% | 41.89% | Exclude |
| 393 | in video / image posts | 44.83% | 50.00% | Exclude |
|  | **Posting a livestream: Young people should not post a livestream of:** |  |  |  |
| 394 | a suicide act | 93.10% | 94.59% | Include |
| 395 | a self-harm act | 93.10% | 95.95% | Include |
|  | **If a young person does livestream content depicting an act of suicide / self-harm, they should:** |  |  |  |
| 396 | turn off the live chat function | 51.72% | 62.16% | Exclude |
| 397 | moderate the live chat function | 34.48% | 79.73% | Exclude |
| 398 | livestream publicly | 0.00% | 6.76% | Exclude |
| 399 | livestream privately | 20.69% | 44.59% | Exclude |
| 400 | share their location with viewers | 41.38% | 43.24% | Exclude |
| 401 | they should not advertise the livestream | 82.76% | 89.19% | Include |
|  | **Responding to a livestream: If a young person comes across a livestream of suicide / self-harm, they should:** |  |  |  |
| 402 | respond with care and compassion | 75.86% | 93.24% | Rerate |
| 403 | provide information, advice and resources | 68.97% | 91.89% | Rerate |
| 404 | report the post to the platform immediately | 100.00% | 91.89% | Include |
| 405 | take recordings / screenshots of content | 24.14% | 33.78% | Exclude |
| 406 | contact police | 82.76% | 85.14% | Include |
| 407 | attempt to save the person themselves if possible and if safe to do so (e.g. go to their location) | 20.69% | 45.95% | Exclude |
| 408 | track the IP address of the person to locate them | 31.03% | 35.14% | Exclude |
| 409 | mobilise others online to track the person down (e.g. find phone details, address, etc.) | 34.48% | 40.54% | Exclude |
| 410 | reach out to other social media users for help | 27.59% | 45.95% | Exclude |
| 411 | if appropriate, contact the person’s family / friends to notify them | 82.76% | 95.95% | Include |
|  | **If a young person comes across a livestream of suicide / self-harm, they should not:** |  |  |  |
| 412 | continue watching the livestream | 72.41% | 63.51% | Exclude |
| 413 | take recordings / screenshots of content | 51.72% | 40.54% | Exclude |
| 414 | express panic or shock | 58.62% | 50.00% | Exclude |
| 415 | make cynical or indifferent comments (e.g. “not again”) | 96.55% | 87.84% | Include |
| 416 | encourage or incite suicide / self-harm | 100.00% | 94.59% | Include |
| 417 | 'like’ or reinforce suicide / self-harm behaviour | 100.00% | 94.59% | Include |
|  | **If a young person who is a considered to be an ‘influencer’ is posting, or receiving messages about, suicide and self-harm, they should:** |  |  |  |
| 418 | be clear about their boundaries and limitations | 100.00% | 97.30% | Include |
| 419 | provide information about sources of support / resources | 96.55% | 97.30% | Include |
| 420 | be clear about their position as an influencer (e.g. that they are not a mental health professional) | 100.00% | 100.00% | Include |
| 421 | consider the impact of their posts on their audience | 96.55% | 98.65% | Include |
| 422 | consider who their audience are when posting about suicide / self-harm (e.g. the age of their followers) | 93.10% | 100.00% | Include |
| 423 | make visible on their page who is managing the page | 79.31% | 77.03% | Rerate |
| 424 | make visible on their page whether comments are being moderated or not | 79.31% | 79.73% | Rerate |
| 425 | make visible on their page whether people can expect an individual/personal response or not | 86.21% | 87.84% | Include |
| 426 | give mental health advice | 44.83% | 41.89% | Exclude |
|  | **If a young person who is a considered an ‘influencer’ is posting, or receiving messages about, suicide and self-harm, they should not:** |  |  |  |
| 427 | portray themselves as an expert in suicide prevention | 89.66% | 86.49% | Include |

**Delphi questionnaire Round 2 results.**

| **Q #** | **Question** | **Pro** | **YP** | **Item result** | **Item number round 1** |
| --- | --- | --- | --- | --- | --- |
| 1 | Young people should communicate (post or respond) about suicide and self-harm without worrying about the potential impact on and consequences for others (i.e., say whatever they want to say without referring or adhering to guidelines). | 4.17% | 19.05% | Exclude | New item |
|  | **If posting about suicide / self-harm, young people should: (Please note, this applies to any type of content.)** |  |  |  |  |
| 2 | Allow comments | 33.33% | 51.16% | Exclude | New item |
| 3 | Always turn off the comments function | 8.70% | 9.30% | Exclude | New item |
| 4 | Turn off the comment function only if the comments will not/can not be monitored | 60.87% | 88.37% | Rerate | New item |
| 5 | If comments are allowed, monitor the post regularly for unsafe or potentially harmful comments | 66.67% | 88.37% | Rerate | 1 |
| 6 | If comments are unmonitored (or turned off), include phone numbers or links to appropriate help services in the post (e.g., helplines, local suicide prevention services, local emergency services if appropriate) | 83.33% | 95.35% | Include | 2 |
|  | **Young people experiencing a current suicidal crisis should:** |  |  |  |  |
| 7 | Post about their thoughts and feelings if they want help as it allows for the possibility of an intervention | 58.33% | 67.44% | Exclude | New item |
| 8 | Post publicly if they want help | 41.67% | 44.19% | Exclude | New item |
| 9 | Send private/direct messages to familiar others online if they want help (friends/followers/community members etc.) | 83.33% | 83.72% | Include | New item |
| 10 | Post about their thoughts and feelings regardless of whether they want help | 0.00% | 9.30% | Exclude | New item |
| 11 | Post publicly if they want help | 8.33% | 11.63% | Exclude | New item |
| 12 | Send private/direct messages to anyone if they want help (friends/followers/community members etc.) | 4.17% | 11.63% | Exclude | New item |
|  | **If a young person intends to post about their thoughts and feelings while in suicidal crisis:** |  |  |  |  |
| 13 | They should draft their post but delay publishing their post (i.e., pressing post). | 12.50% | 60.47% | Exclude | New item |
|  | **If a young person is sharing their own thoughts or experiences of suicide, they should:** |  |  |  |  |
| 14 | Include information on support services (e.g., links to suicide prevention services or counselling helpline) | 79.17% | 90.70% | Rerate | 19 |
|  | **If a young person is sharing their own thoughts or experiences of self-harm, they should:** |  |  |  |  |
| 15 | Include information on support services (e.g., links to suicide prevention services or counselling helpline) | 83.33% | 97.62% | Include | 27 |
|  | **If a young person is sharing their own thoughts or experiences of suicide, they should not:** |  |  |  |  |
| 16 | Include content that makes others feel responsible for their safety | 54.17% | 86.05% | Rerate | 36 |
| 17 | Plan their own suicide or self-harm on social media | 83.33% | 95.35% | Include | 38 |
|  | **If a young person is sharing their own thoughts or experiences of self-harm, they should not:** |  |  |  |  |
| 18 | Include content that makes others feel responsible for their safety | 58.33% | 88.10% | Rerate | 41 |
| 19 | Plan their own suicide or self-harm on social media | 83.33% | 95.24% | Include | 43 |
|  | **If a young person feels that another person has helped them in terms of their suicide / self-harm, they should thank them:** |  |  |  |  |
| 20 | Privately | 41.67% | 65.12% | Exclude | 60 |
| 21 | If a young person feels that another person has helped them in terms of their suicide / self-harm, they should not thank them | 8.33% | 4.65% | Exclude | New item |
|  | **Young people should not post content that:** |  |  |  |  |
| 22 | Is intended to shock or disgust others | 87.50% | 88.37% | Include | 63 |
| 23 | Provides information about suicide pacts | 100.00% | 90.70% | Include | 66 |
| 24 | Includes suggestive signs or emojis that suggest suicide / self-harm (e.g., methods of self-harm / suicide) | 83.33% | 76.74% | Rerate | 67 |
| 25 | Contains a suicide note or message | 79.17% | 74.42% | Rerate | 70 |
|  | **Young people should not share content that:** |  |  |  |  |
| 26 | Is intended to shock or disgust others | 87.50% | 88.10% | Include | 73 |
| 27 | Provides information about suicide pacts | 100.00% | 88.10% | Include | 76 |
| 28 | Includes suggestive signs or emojis that suggest suicide / self-harm (e.g., methods of self-harm / suicide) | 83.33% | 80.95% | Include | 77 |
| 29 | Contains a suicide note or message | 79.17% | 73.81% | Rerate | 80 |
|  | **If a young person is sharing their thoughts or experiences of suicide / self-harm because they want help, they should:** |  |  |  |  |
| 30 | Explicitly state that they need support | 95.83% | 93.02% | Include | 92 |
|  | **If a young person is sharing their thoughts or experiences of suicide / self-harm but they do not want help, they should:** |  |  |  |  |
| 31 | Explicitly state that they are expressing their thoughts, feelings and experiences simply to make themselves feel better (i.e., venting) | 66.67% | 76.74% | Exclude | 95 |
|  | **Young people should provide an accompanying trigger or content warning to:** |  |  |  |  |
| 32 | Any post relating to suicide / self-harm (e.g., even if there are no images) | 66.67% | 95.35% | Rerate | 108 |
|  | **When posting a warning, young people should use the following language:** |  |  |  |  |
| 33 | Trigger warning' | 50.00% | 88.37% | Rerate | 99 |
| 34 | Content warning' | 79.17% | 95.35% | Rerate | 101 |
|  | **When posting a warning, young people should include (i.e., place) the warning:** |  |  |  |  |
| 35 | In the first image of the content (e.g. in the case of a series of images being posted) | 79.17% | 93.02% | Rerate | 113 |
| 36 | Information on what is contained in the post (e.g., "warning - this post contains information on suicide / self-harm / images of somebody who has died by suicide") | 79.17% | 95.35% | Rerate | 115 |
| 37 | The option for users to view the content anyway, even if there is a warning (e.g., being able to accept the warning and click through to the post) | 43.48% | 90.48% | Rerate | 116 |
| 38 | Young people should add a 'mature content' (18+) warning or tag to all suicide / self-harm related content. | 20.83% | 44.19% | Exclude | New item |
|  | **Young people should not post or share images, photos, video content or animations:** |  |  |  |  |
| 39 | That depict the location of a suicide / self-harm | 91.67% | 81.40% | Include | 123 |
| 40 | Of wounds | 91.67% | 88.37% | Include | 124 |
| 41 | Of injuries | 87.50% | 90.48% | Include | 125 |
| 42 | Of grieving friends or family members of those who have died by suicide without their permission | 95.83% | 95.35% | Include | 130 |
| 43 | Of the deceased person’s body (e.g., photographs from a funeral viewing) | 100.00% | 88.37% | Include | 132 |
| 44 | That blurs images of methods, but includes details of methods in the accompanying text (e.g., in the caption or hastags) | 91.67% | 90.48% | Include | 133 |
|  | **Young people should not post or share videos of:** |  |  |  |  |
| 45 | During a live attempt at suicide - The lead-up to a suicide | 100.00% | 93.02% | Include | 140 |
| 46 | During a live attempt at suicide - Suicide attempts in progress | 87.50% | 95.35% | Include | 141 |
| 47 | When someone has already died by suicide - The lead-up to a suicide | 95.83% | 79.07% | Rerate | 143 |
| 48 | When someone has already died by suicide - Suicide attempts in progress | 95.83% | 95.35% | Include | 144 |
| 49 | If posting images / photos / videos relating to suicide / self-harm, young people should use captions (e.g. text under the visual content) to provide context | 50.00% | 83.72% | Rerate | 146 |
|  | **When posting about suicide, young people should not use language that:** |  |  |  |  |
| 50 | Describes suicide as criminal or sinful (e.g., committed suicide) | 95.83% | 93.02% | Include | 149 |
|  | **If a young person is concerned about someone who has experienced suicidal or self-harm thoughts or feelings, they should not:** |  |  |  |  |
| 51 | Post anything they would not say directly to them in-person | 75.00% | 95.35% | Rerate | 157 |
| 52 | Post anything without their permission | 54.17% | 97.67% | Rerate | 158 |
|  | **If writing or sharing a post about someone who has died by suicide, young people should:** |  |  |  |  |
| 53 | Death of someone the young person knows - Dispel myths or misinformation | 83.33% | 83.72% | Include | 163 |
| 54 | Death of someone the young person knows - Explicitly state that they have received permission from the family in their post | 45.83% | 74.42% | Exclude | 164 |
| 55 | Death of someone the young person knows - Moderate comments | 54.17% | 90.70% | Rerate | 167 |
| 56 | Death of someone the young person knows - Be clear about whether people can contact them for support | 79.17% | 93.02% | Rerate | 168 |
| 57 | Death of someone the young person knows - Obtain permission from the deceased’s family | 50.00% | 88.37% | Rerate | New item |
| 58 | Death of someone the young person knows - Discuss why they think someone has died by suicide but without assigning blame on other people or single events. This is to allow for sense making, mutual support, and facilitation of the grieving process. | 29.17% | 44.19% | Exclude | New item |
| 59 | Death of a celebrity or public figure - Post only what they know to be true | 100.00% | 100.00% | Include | 173 |
| 60 | Death of a celebrity or public figure - Moderate comments | 33.33% | 74.42% | Exclude | 181 |
| 61 | Death of a celebrity or public figure - Be clear about whether people can contact them for support | 79.17% | 83.72% | Rerate | 182 |
|  | **If writing or sharing a post about someone who has died by suicide, young people should not:** |  |  |  |  |
| 62 | Death of someone the young person knows - Speculate or form theories about a person’s thoughts or feelings leading up to the suicide | 83.33% | 90.70% | Include | 188 |
| 63 | Death of someone the young person knows - Speculate or form theories about why the person took their life | 82.61% | 88.37% | Include | 189 |
| 64 | Death of someone the young person knows - Allow comments that indicate the poster is going to attempt suicide (e.g., 'I will join you soon') | 66.67% | 95.35% | Rerate | 195 |
| 65 | Death of someone the young person knows - Share content that discusses the suicide in an unsafe way (e.g., describes methods, contains graphic images) | 100.00% | 95.35% | Include | 200 |
| 66 | Death of a celebrity or public figure - Speculate or form theories about a person’s thoughts or feelings leading up to the suicide | 83.33% | 93.02% | Include | 203 |
| 67 | Death of a celebrity or public figure - Speculate or form theories about why the person took their life | 87.50% | 88.37% | Include | 204 |
| 68 | Death of a celebrity or public figure - Post or share information that they are unsure of or that they know is inaccurate | 100.00% | 95.35% | Include | 205 |
| 69 | Death of a celebrity or public figure - Share content that discusses the suicide in an unsafe way (e.g., describes methods, contains graphic images) | 100.00% | 97.67% | Include | 215 |
| 70 | Death of a celebrity or public figure - Post or share too many posts about the suicide back-to-back | 79.17% | 81.40% | Rerate | 216 |
|  | **If a young person comes across a post that shares a suicide story in a way they consider to be unsafe, they should:** |  |  |  |  |
| 71 | Encourage the poster to reword their message so it is safer and repost | 66.67% | 79.07% | Exclude | 218 |
|  | **If a young person comes across content that suggests a person may be thinking about suicide or self-harm, they should:** |  |  |  |  |
| 72 | Seek professional advice (e.g., a phone or online support service, or health professional) | 79.17% | 97.67% | Rerate | 223 |
|  | **If a young person comes across content that suggests a person may be thinking about suicide or self-harm, they should not:** |  |  |  |  |
| 73 | Share the post | 70.83% | 90.70% | Rerate | 229 |
| 74 | Say that the person is an "attention seeker" | 95.83% | 93.02% | Include | New item |
|  | **If the person is not at immediate risk of suicide, young people should:** |  |  |  |  |
| 75 | Ask the person "what can I do to help?" | 75.00% | 83.72% | Rerate | 232 |
|  | **If the person is at immediate risk of suicide, young people should:** |  |  |  |  |
| 76 | Call emergency services immediately (and if appropriate tell the person they’re doing this) | 83.33% | 88.37% | Include | 236 |
| 77 | Contact appropriate emergency services in their country (e.g., ambulance rather than police) | 79.17% | 90.70% | Rerate | New item |
|  | **The following items relate to additional things that young people could do if they want to interact with the person posting. If a young person decides to respond, they should:** |  |  |  |  |
| 78 | Tell the person that they care | 91.67% | 95.35% | Include | 249 |
| 79 | Respond using the medium that they (the responder) feels most comfortable with (e.g., messages, comment, phone call) | 91.67% | 97.50% | Include | 254 |
| 80 | Look at the person’s posts to acknowledge their feelings and specify exactly why they are worried about them | 83.33% | 83.72% | Include | 255 |
| 81 | Give the person their full attention (if engaged in conversation) | 75.00% | 90.48% | Rerate | 256 |
| 82 | Try and assess the level of risk (e.g., ask if they have a suicide / self-harm plan, intent, and/or means) | 12.50% | 67.44% | Exclude | 257 |
| 83 | Cautiously check whether the person has further suicide plans | 41.67% | 81.40% | Rerate | 260 |
| 84 | Offer to ‘sit’ with the person virtually in real time | 58.33% | 88.37% | Rerate | 264 |
| 85 | Seek and obtain formal or informal support for themselves | 75.00% | 92.86% | Rerate | New item |
|  | **If a young person decides to respond to a post that concerns them, they should not:** |  |  |  |  |
| 86 | Tag others in their reply | 75.00% | 81.40% | Rerate | 268 |
| 87 | Offer support if they do not feel able or comfortable to do so | 100.00% | 95.35% | Include | 271 |
| 88 | Respond alone (i.e., have someone else with them, or inform someone else) | 37.50% | 39.53% | Exclude | New item |
| 89 | If a young person experiences a user/account as unhelpful or harmful, they should mute or unfollow the account/user rather than delete or block them. This is to protect the consumer from being exposed to things that they do not want to see, and protects the creator from feeling rejected and abandoned. | 82.61% | 86.05% | Include | New item |
|  | **Suicide / self-harm groups or account moderators should:** |  |  |  |  |
| 90 | Be an adult (18+) | 66.67% | 83.72% | Rerate | New item |
| 91 | Be supported and supervised by an adult | 75.00% | 93.02% | Rerate | New item |
|  | **If running a closed group or forum, moderators should:** |  |  |  |  |
| 92 | Remove or suspend members that violate terms of use | 83.33% | 93.02% | Include | 283 |
| 93 | Mute users who violate 'Terms of Use' | 86.36% | 88.10% | Include | New item |
| 94 | Group/account membership should not be contingent on current suicide / self-harm thoughts, feelings, or behaviour. | 83.33% | 74.42% | Rerate | New item |
|  | **If setting up a closed group or forum, young people should not:** |  |  |  |  |
| 95 | Require group members to share evidence of suicide / self-harm as part of their application to join the group | 100.00% | 97.67% | Include | 291 |
|  | **If creating a group or account to memorialise somebody who has died by suicide, young people should:** |  |  |  |  |
| 96 | Make it clear to users that there is likely to be detailed information and images of people who have died | 58.33% | 97.67% | Rerate | 311 |
| 97 | Host the memorial/commenoration in collaboration with the deceased’s family | 41.67% | 74.42% | Exclude | New item |
| 98 | Young people should exit pro-suicide or pro-self-harm groups (i.e., those that deliberately or inadvertently encourage and promote suicide or self-harm). | 75.00% | 97.67% | Rerate | New item |
|  | **A suicide hoax is a deliberate report of somebody’s death that is later proven to be untrue. Young people should:** |  |  |  |  |
| 99 | Report the hoax to e-Safety Commissioner or similar government body | 95.83% | 93.02% | Include | 332 |
|  | **A suicide hoax is a deliberate report of somebody’s death that is later proven to be untrue. Young people should not:** |  |  |  |  |
| 100 | Share or re-post content about suicide hoaxes | 95.83% | 92.86% | Include | 334 |
| 101 | Interact with posts about suicide hoaxes (e.g., comment, like or react) | 83.33% | 90.48% | Include | 335 |
|  | **When responding to a post about self-harm, young people should:** |  |  |  |  |
| 102 | Suggest alternatives to self-harm (e.g., self-soothing strategies, a virtual hope box) | 83.33% | 88.37% | Include | 351 |
| 103 | Suggest distractions from self-harm (e.g., exercise, taking a hot bath, listening to soothing music) | 87.50% | 88.37% | Include | 352 |
| 104 | Suggest wider coping strategies to deal with the underlying problem/s (e.g., speak to friends) | 91.67% | 97.67% | Include | 353 |
| 105 | Positively reinforce and praise milestones and progress of others | 95.83% | 90.48% | Include | 355 |
| 106 | Remind people that ‘lapses’ or ‘relapses’ can be part of the process | 91.67% | 95.35% | Include | 356 |
| 107 | Encourage the person to keep trying to refrain from self-harm if they have lapsed or relapsed | 87.50% | 92.86% | Include | 357 |
| 108 | Post educational information to help people who are self-harming (e.g., how they can stop) | 83.33% | 90.70% | Include | 367 |
|  | **When either posting about, or responding to posts about, self-harm, young people should not:** |  |  |  |  |
| 109 | Normalise self-harm behaviour (e.g., post that self-harm is an acceptable coping behaviour, fixes things or makes you feel better) | 100.00% | 93.02% | Include | 368 |
| 110 | Post advice on how people can self-harm without injuring themselves too badly | 75.00% | 74.42% | Rerate | 374 |
|  | **Young people should not use humour in these circumstances:** |  |  |  |  |
| 111 | When someone has died by suicide | 83.33% | 86.05% | Include | 390 |
| 112 | When someone has self-harmed | 79.17% | 86.05% | Rerate | 391 |
|  | **If a young person comes across a livestream of suicide / self-harm, they should:** |  |  |  |  |
| 113 | Respond with care and compassion | 79.17% | 92.86% | Rerate | 402 |
| 114 | Provide information, advice, and resources | 66.67% | 85.71% | Rerate | 403 |
|  | **If a young person who is a considered to be an ‘influencer’ is posting, or receiving messages about, suicide and self-harm, they should:** |  |  |  |  |
| 115 | Make visible on their page who is managing the page | 75.00% | 72.09% | Rerate | 423 |
| 116 | Make visible on their page whether comments are being moderated | 95.83% | 93.02% | Include | 424 |
| 117 | Only share evidence-based suicide / self-harm resources | 83.33% | 90.70% | Include | New item |
| 118 | Consult with mental health professionals before, during, and after communications related to suicide / self-harm | 62.50% | 88.37% | Rerate | New item |
